# Expression of alternative oxidase in *Drosophila* ameliorates diverse phenotypes due to cytochrome oxidase deficiency

**DOI:** 10.1093/hmg/ddt601

**Published:** 2013-11-29

**Authors:** Kia K. Kemppainen, Juho Rinne, Ashwin Sriram, Matti Lakanmaa, Akbar Zeb, Tea Tuomela, Anna Popplestone, Satpal Singh, Alberto Sanz, Pierre Rustin, Howard T. Jacobs

**Affiliations:** 1 Institute of Biomedical Technology and Tampere University Hospital, University of Tampere, FI-33014 Tampere, Finland,; 2 School of Medicine and Biomedical Sciences, State University of New York at Buffalo, 206 Cary Hall, Buffalo, NY 14214, USA,; 3 INSERM UMR 676, Hôpital Robert Debré, 48 Bd Sérurier, 75019 Paris, France and; 4 Molecular Neurology Research Program, University of Helsinki, FI-00014 Helsinki, Finland

## Abstract

Mitochondrial dysfunction is a significant factor in human disease, ranging from systemic disorders of childhood to cardiomyopathy, ischaemia and neurodegeneration. Cytochrome oxidase, the terminal enzyme of the mitochondrial respiratory chain, is a frequent target. Lower eukaryotes possess alternative respiratory-chain enzymes that provide non-proton-translocating bypasses for respiratory complexes I (single-subunit reduced nicotinamide adenine dinucleotide dehydrogenases, e.g. Ndi1 from yeast) or III + IV [alternative oxidase (AOX)], under conditions of respiratory stress or overload. In previous studies, it was shown that transfer of yeast Ndi1 or *Ciona intestinalis* AOX to *Drosophila* was able to overcome the lethality produced by toxins or partial knockdown of complex I or IV. Here, we show that AOX can provide a complete or substantial rescue of a range of phenotypes induced by global or tissue-specific knockdown of different cIV subunits, including integral subunits required for catalysis, as well as peripheral subunits required for multimerization and assembly. AOX was also able to overcome the pupal lethality produced by muscle-specific knockdown of subunit CoVb, although the rescued flies were short lived and had a motility defect. cIV knockdown in neurons was not lethal during development but produced a rapidly progressing locomotor and seizure-sensitivity phenotype, which was substantially alleviated by AOX. Expression of Ndi1 exacerbated the neuronal phenotype produced by cIV knockdown. Ndi1 expressed in place of essential cI subunits produced a distinct residual phenotype of delayed development, bang sensitivity and male sterility. These findings confirm the potential utility of alternative respiratory chain enzymes as tools to combat mitochondrial disease, while indicating important limitations thereof.

## INTRODUCTION

Mitochondrial diseases affecting the respiratory complexes of the oxidative phosphorylation (OXPHOS) system are a diverse collection of pathologies, which can affect almost any tissue, at any age (1,2). They are typically progressive in nature, and no effective treatments are currently available. Where genetic causes are known, they can include lesions in any of hundreds of genes whose products are needed for the biosynthesis or function of the respiratory complexes. These genes, moreover, are distributed between nuclear and mitochondrial DNA (mtDNA), and a subset of mitochondrial diseases also results from defective communication between the cell's two genomes.

In order to understand better the pathophysiological mechanisms of mitochondrial disease, and develop a possible strategy for eventual therapy, we have exploited the fact that lower eukaryotes, including plants and many invertebrates, possess an alternative, non-proton-pumping respiratory chain in mitochondria, whose components can act as a bypass of the OXPHOS system under conditions of respiratory stress or overload (3,4). Alternative reduced nicotinamide adenine dinucleotide(NADH) dehydrogenases such as yeast Ndi1 can replace complex I (cI), while alternative oxidases (AOXs) can replace complexes III + IV (cIII + IV). The alternative enzymes are each composed of a single gene product, which together conduct electrons from NADH to molecular oxygen via ubiquinone as an intermediate electron acceptor. AOX becomes enzymatically active only when electron transfer via the OXPHOS system becomes inhibited beyond ubiquinone. The main mechanism to achieve this is believed to be that AOX has a much higher *K*_m_ for its substrate, ubiquinol, than does cIII. In contrast, Ndi1 may be constitutively active, but its role in electron flow under normal physiological conditions may be limited by the tight coupling between cI and cIII in supercomplexes.

Like Ndi1, AOX is absent from vertebrates, as well as arthropods (5), but we reasoned that if the gene product were transferred to higher metazoans it might be able to functionally replace the corresponding OXPHOS complexes under conditions resembling those pertaining in mitochondrial disease. When expressed transgenically in human cells (6,7), *Drosophila* (8) or most recently the mouse (9), AOX from the urochordate *Ciona intestinalis* was shown to be enzymatically functional when cIII or cIV was inhibited by specific toxins, or genetic mutation. Similarly, when yeast Ndi1 was transferred to mammalian cells or to *Drosophila*, it was functional, enabling NADH oxidation when cI was inhibited by rotenone (10). Remarkably, the ubiquitous presence of these alternative enzymes is tolerated by the whole organism, to which it can confer toxin resistance *in vivo* (8–10). In wild-type flies, no deleterious phenotype is produced by constitutive AOX (or Ndi1) expression (8,10). However, the alternative enzymes can overcome the lethality produced by partial knockdown of at least some subunits of the corresponding OXPHOS complexes, including two subunits of the membrane portion of cI (10), a nuclear-coded ‘supernumerary’ subunit of cIV [Cox6c, the *cyclope* gene product (8)] and a cIV assembly factor, Surf1 (8). Note, however, that AOX cannot complement the total loss of cIV, e.g. via a null mutation in Cox6c (9).

The structure of metazoan cIV is relatively well understood (11). It comprises a catalytic core, composed of the three mtDNA-encoded subunits, whose functional assembly is dependent on a set of phylogenetically conserved nuclear-coded subunits (Cox4, Cox5a, Cox5b, Cox6b, Cox6c, probably Cox7c, Cox8, using the mouse nomenclature) required to produce, via a stepwise assembly pathway, a membrane-bound sub-complex with enzymatic activity (12,13). The incorporation of prosthetic groups (*a*-type haemes and copper) is also involved in this process. The other nuclear-coded subunits (Cox6a and Cox7a) are then incorporated to form the fully functional cIV, which also contains a recently described subunit (14) formerly believed to be a constituent of cI. Some of these subunits, notably Cox6a, are believed to be important for dimerization of the complex and, together with other specific proteins such as Rcf1 (HIG2A) and Cox7RP, may also be required for the formation of supercomplexes containing cIV, as well as cI and/or cIII in different stoichiometries (15–18). The more specific roles of the nuclear-coded subunits of cIV (often termed ‘supernumerary’ since they are absent in bacteria), for example in physiological regulation, are less clear. Null mutations in most of the above subunits are lethal in *Drosophila*, whereas a splice-site mutation in the *levy* gene, encoding subunit Cox6a, produces an adult-onset neurodegenerative phenotype (19).

Cytochrome *c* oxidase (COX) deficiency in humans has a wide variety of pathological manifestations and a diversity of genetic causes (20,21). Organs affected can be the central nervous system, skeletal or heart muscle, the liver or a combination of these and other organs, and tissue-specificity is poorly understood. Underlying genetic defects commonly impact accessory factors for cIV biosynthesis, including assembly factors such as SURF1 (22,23) or C2ORF64 (24) or proteins involved in cofactor synthesis or transport, such as SCO2 (25) and COX10 (26). Comparatively rare mutations are also found in genes for structural subunits of cIV, such as COX6B1 (27) or COX7B (28), as well as those encoded in mtDNA (29–31), plus genes required mainly or exclusively for the biosynthesis of mtDNA-encoded cIV subunits [e.g. LRPPRC (32), TACO1 (33)]. Pathological COX deficiency has been reported in many other disorders (34,35), including neurodegenerative conditions such as Alzheimer dementia (36–38) and Huntington's disease (39) although its aetiological significance remains unclear. COX is also a pathological target in ischaemia, sepsis and other types of toxic injury (40).

Some cIV subunits are encoded by isogenes differentially expressed between tissues, where they are adapted to specific physiological conditions (35,41). Tissues also vary in their substrate dependence, reflected in the degrees to which the different OXPHOS complexes contribute to threshold effects for respiration (42). Furthermore, supercomplexes, whose formation may also vary between tissues, entrain a greater or lesser degree of channelling of respiratory electron flow, and may also limit the degree to which exogenously introduced alternative respiratory enzymes can contribute to respiration (18). In a general sense, these phenomena are believed to contribute to the bewildering tissue-diversity of mitochondrial disease, though there remain few concrete mechanistic explanations for this. The relative importance in COX-associated pathology of disturbed redox homeostasis, apoptosis induction, deranged cell signalling, adenosine triphosphate (ATP) deficiency, proteotoxic stress and other types of metabolic disturbance remains a topic of intense debate.

The possible use of AOX in future therapies for COX deficiency will obviously be limited by these considerations, as well as by the fact that the alternative respiratory chain enzymes do not contribute directly to ATP production. Completely replacing the function of one or more OXPHOS complexes cannot completely restore ATP production capacity to wild-type, even though it can facilitate proton-pumping linked to electron transfer at OXPHOS complexes other than the one(s) it is bypassing. Because AOX bypasses two of the three proton-translocating steps, it should not restore ATP production for those substrates whose oxidation supplies electrons directly to ubiquinone via cII or other dehydrogenases. Even if AOX can restore redox balance, mitigate mitochondrial reactive oxygen species (ROS) production (6–9) and limit systemic lactic acidosis (6), tissues that depend on such substrates may still suffer a substantial loss of function that AOX cannot correct. On the other hand, where COX activity is limiting for respiration, AOX may promote a significant restoration of ATP production (via proton pumping at other sites), as well as other metabolic benefits.

*Drosophila* offers a convenient model for human diseases, including those affecting mitochondrial functions. The composition of the OXPHOS complexes, the overall structure and gene content of mtDNA and the energetic physiology of the main organ systems are all similar to those of humans. The fact that AOX expression in the fly is able to confer cyanide resistance and overcome the lethality of partial knockdown of Cox6c or *Surf1* raises the question of how far it is able to replace cIV throughout the life cycle. We thus set out to use the fly as a model system to address this issue, focusing on three specific questions relevant to understanding the pathophysiology of mitochondrial disease and the possible future use of AOX in therapy. First, what phenotypes result from deficiencies in different classes of cIV subunits, and can AOX alleviate these phenotypes? Secondly, in which tissues is loss of COX function crucial for producing such phenotypes, and their rescue by AOX? Thirdly, what residual phenotypes result from replacing cIV function with AOX? The many genetic tools available in *Drosophila* (including effective and specific RNAi *in vivo*) make it an ideal tool for such a study, in advance of translating the most prominent findings to the mouse, which is less flexible, and also takes much longer and is substantially more expensive to manipulate.

In a first step, to validate use of the inducible (upstream activating sequence) UAS-AOX transgenic lines that we created previously, we first conducted an important control to verify that the rescue seen previously was not subject to any confounding, ‘promoter-dilution’ effects from the simultaneous use of transgenes dependent on the same transcription factor. We then proceeded to test the phenotypes generated by knockdown of different cIV subunits in the whole fly and in specific tissues, and the ability of AOX to rescue these phenotypes, focusing on the major differentiated cell-types affected by human mitochondrial disease (muscle and neurons).

The results highlight the different phenotypes produced by deficiencies of different subunits, indicate a crucial developmental role for cIV in muscle and confirm COX deficiency as a cause of adult-onset neurodegeneration. AOX expression was able to partially rescue these phenotypes. In contrast, loss of proton-pumping at cI promotes a distinct phenotype of developmental delay, bang sensitivity and male infertility.

## RESULTS

### Constitutive low-level AOX expression confers resistance to COX inhibition

**Figure 1.**
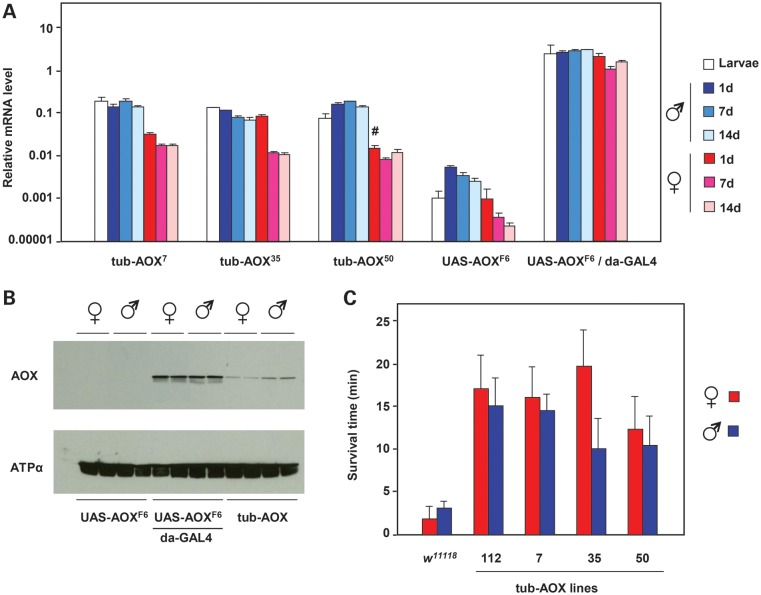
Transgenic expression of *tub-AOX*. (**A**) Quantitative real time-polymerase chain reaction (qRT-PCR) analysis of AOX mRNA expression (normalized against *RpL32*) in transgenic adults and larvae, as indicated. Means ± SD of at least three technical replicates of each of at least three biological replicates. Comparing *(upstream activating sequence) UAS-AOX* expression driven by *da-GAL4* (yeast transcription activator protein GAL4) with that of *tub-AOX*, *P* < 0.01 for each sex/age analysed; similarly, comparing *UAS-AOX* expression in the absence of driver with that of *tub-AOX*, *P* < 0.01 in each case, except where indicated (#), where *P* < 0.05 (Student's *t* test, two-tailed, unequal variances). (**B**) Western blot of AOX protein and adenosine triphosphate (ATP) synthase subunit α (loading control) in 1-day-old adults of the transgenic strains indicated. tub-AOX denotes flies homozygous for each of *tub-AOX^7^*, *tub-AOX^35^* and *tub-AOX^50^* transgenes (males are hemizygous for *tub-AOX^35^*). Replicate batches of protein extracts from 30 females or 40 males of each genotype are shown in adjacent lanes. (**C**) Survival time on cyanide-impregnated agar of flies of the (homozygous) strains indicated. Means ± SD of 80–100 flies of each group, in batches of 10 flies per vial. *P* < 0.01 in each case, in comparison with *w^1118^* control flies of same sex (Student's *t* test, two-tailed, unequal variances). See also Supplementary Material, Figure S1.

In previous experiments we expressed inducible AOX using the UAS/Gal4 (yeast transcription activator protein GAL4) system, which partially rescued the lethality of cyanide treatment or the disruption of COX by knockdown of the Cox6c subunit (the *cyclope* gene: see Table 1) or the Surf1 assembly factor. However, the UAS/Gal4 system results in a very high level of transgene expression. Furthermore, since COX knockdown by RNAi also involves the same induction system, we cannot completely exclude a contribution to the rescue from promoter competition, even though UAS-GFP (green fluorescent protein) was unable to rescue. We therefore created transgenic flies bearing the same AOX transgene, but under the control of the constitutive α-tubulin promoter (*tub*-AOX), with independent single insertions into non-coding DNA on chromosomes X, 2 and 3 (Supplementary Material, Fig. S1A–D). Expression of these transgenes at the RNA level was 20–50-fold lower than from UAS-AOX driven by *da*-GAL4 (Fig. 1A), though still much higher than UAS-AOX in the absence of a GAL4 driver. *tub*-AOX expression was substantially higher in larvae and adult males than females (Fig. 1A), and was maintained over the first 2weeks of adult life to a variable extent.

**Table 1.** Nomenclature, expression patterns and assembly of COX subunits in *Drosophila*

Subunit name^a^	Official gene name(s) and symbol(s)^b^	Expression pattern(s)^c^	Comments^d^
Cox1	mt CoI	Ubiquitous	mtDNA-encoded, part of core sub-complex S2
Cox2	mt CoII	Ubiquitous	mtDNA-encoded, incorporated into sub-complex S3
Cox3	mt CoIII	Ubiquitous	mtDNA-encoded, incorporated into sub-complex S3
Cox4	*CoIV*	Ubiquitous	Two isogenes with different expression patterns, part of core sub-complex S2
	*CG10396*	Testis-specific	
Cox5a	*CoVa*	Ubiquitous	Part of core sub-complex S2
Cox5b	*CoVb*	Ubiquitous	Incorporated into sub-complex S3
Cox6a	*levy*	Ubiquitous	Incorporated at final assembly steps into mature complex IV; *levy^1^* splice-site mutant manifests adult-onset neurodegeneration (19)
Cox6b	*CoVIb*	Ubiquitous	Incorporated into sub-complex S3
Cox6c	*cyclope*	Ubiquitous	Null-mutant larval lethal, incorporated into sub-complex S3
Cox7a	*CG9603*	Ubiquitous, lowest in testis	Incorporated at final assembly steps into mature complex IV; 99% of brain Cox7a expression contributed by CG9603
	*CG34172*	Mainly muscle-specific (heart crop, hindgut, carcass, lower in head)	
	*CG18193*	Testis-specific	
Cox7b	None identified		Proposed to be required for an early assembly step (28); clear orthologues identified only in vertebrates
Cox7c	*CoVIIc*	Ubiquitous, but low in testis	Only recently identified in *Drosophila*
Cox8	*CoVIII*	Ubiquitous, but low in testis	Incorporated into sub-complex S3

^a^Using mouse nomenclature. Note that, some subunits are encoded by gene families in mouse but single genes in *Drosophila*, and *vice versa*.

^b^From www.flybase.org.

^c^From www.flyatlas.org.

^d^Assembly program based on Ref. (12).

AOX expression at the protein level was also less than when driven by GAL4, even when we combined *tub*-AOX insertions on all three major chromosomes into a single line of flies (Fig. 1B). *tub*-AOX conferred cyanide resistance to substrate oxidation by isolated mitochondria (Supplementary Material, Fig. S1E), and single *tub*-AOX insertions were able to confer almost as great a time of resistance to cyanide as UAS-AOX driven by da-GAL4 (Fig. 1C, compare with Fig. 5A of Ref. 8).

**Figure 2.**
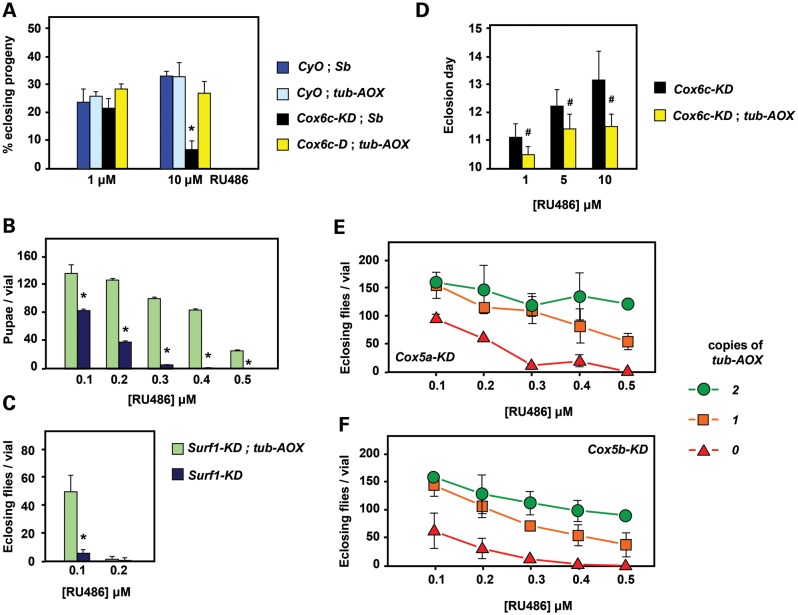
Rescue of COX deficiency by *tub-AOX*. (**A**) *tub-AOX* rescue of developmental lethality and (**D**) of developmental delay, from partial knockdown of Cox6c (*Drosophila* gene *cyclope*) using *tub-GS* driver. Proportion of eclosing progeny or eclosion day for different genotypes and concentrations of RU486 as shown, means ± SD from 4 or more biological replicates. (**B** and **C**) *tub-AOX* rescue of developmental lethality from partial knockdown of Surf1 using *tub-GS* driver. The number of pupae or eclosing flies for different genotypes and concentrations of RU486 as shown, means ± SD from 3 or more biological replicates. (**E** and **F**) Rescue of developmental lethality from partial knockdown of Cox5a or Cox5b (*Drosophila* genes *CoVa*, *CoVb*) using *tub-GS* driver, as shown. Number of eclosing progeny at different concentrations of RU486, for the indicated numbers of *tub-AOX* transgenes. Means ± SD from 3 or more biological replicates. *P* < 0.01 (*) or < 0.05 (#), Student's *t* test, two-tailed, unequal variances, comparing flies with and without *tub-AOX*. See also Supplementary Material, Figure S2. Note that at 0 μm RU486 all knockdown lines tested were indistinguishable from wild-type flies in the assays shown, and that wild-type flies eclose on Days 10 and 11 at 25°C. Two copies of *tub*-AOX also rescued the lethality of Cox6b knockdown (Supplementary Material, Fig. S2C).

A single copy of *tub-AOX* was also able to rescue the semi-lethality of knockdown of either Cox6c (Fig. 2A) or Surf1 (Fig. 2B and C), when RNAi was induced with different doses of RU486, in the presence of ubiquitously expressed GeneSwitch, a modified version of GAL4 that is dependent on the drug for activity. [Note that, for clarity, we use the mammalian (mouse) names for all subunits and the genes that encode them throughout the text, indicating the *Drosophila* gene name only upon first mention, in Table 1 and in figure legends.] Knockdown of Cox6c by about 80% at the RNA level, using 10 μm RU486 (Supplementary Material, Fig. S2A), not only resulted in lethality of about three-quarters of the progeny (Fig. 2A), but those flies that were able to eclose did so with a 3-day delay (Fig. 2D), a phenotypic feature seen in many *Drosophila* OXPHOS mutants. The presence of *tub-AOX* corrected both of these phenotypes (Fig. 2A and D). *tub-AOX* also enabled developing flies to reach pupal stage at doses of RU486 that induced a degree of Surf1 knockdown that prevented any pupariation of control flies (Fig. 2B). At 0.1 μm of the drug, a substantial number of *tub-AOX* expressors even reached eclosion, whereas only a few viable adults were produced at this dose by control flies under Surf1 knockdown alone (Fig. 2C).

### Extent of COX deficiency rescued by AOX expression

Knockdown of subunits Cox5a or Cox5b also produced an RU486 dose-dependent lethality (Fig. 2E and F). This enabled us to test how rescue is affected by the number of constitutively expressed copies of AOX, and to estimate the amount of knockdown of COX required to produce AOX-rescuable lethality. A single copy of *tub-AOX* was sufficient to overcome the lethality of knocking down Cox5a or Cox5b to about half of the wild-type level at larval stage L3 (Supplementary Material, Fig. S2A), which also produced a decrease of about 50% in the enzymatically measured COX activity (Supplementary Material, Fig. S2B). Flies rescued from the lethality of Cox5a or Cox5b knockdown by two copies of *tub-AOX* eclosed with a slight, but statistically non-significant developmental delay (Supplementary Material, Fig. S2D). The rescued flies were fertile when mated to wild-type flies of the opposite sex.

Flies in which Cox5b was knocked down during development by a higher dose (3 μm) of RU486, failed to develop beyond the larval stage, but four copies of *tub-AOX* enabled the flies to reach pupal stage, with 12% of the pupae eclosing. The flies were tested for bang sensitivity, a sensorineural phenotype associated with mitochondrial dysfunction, but no bang sensitivity was found (Supplementary Material, Fig. S2E).

Having excluded promoter-dilution effects as a reason for AOX rescue of COX deficiency, we set out to use the UAS-AOX lines, in combination with different GAL4 drivers, to analyse the developmental phenotypes produced by knockdown of COX subunits, and the degree to which AOX expression is able to complement them.

### AOX expression rescues developmental lethality of COX knockdown

The nuclear-coded subunits of cIV are believed to confer various regulatory properties on COX, as well as being required for its proper assembly and stability. Deficiency of specific subunits leads to the accumulation of various assembly intermediates and to subtly different phenotypes. Most nuclear-coded subunits of COX in *Drosophila* are encoded by single-copy nuclear genes (Table 1). Two notable exceptions are Cox4, encoded by a specific isogene (CG10396) in testis, with a second isogene (*CoIV*, CG10664) expressed ubiquitously, and subunit Cox7a, also encoded by testis-specific (CG18193) and ubiquitous (CG9603) isogenes, plus an additional, widely expressed isogene (CG34172), prominently expressed in muscle (carcass, crop, hindgut and heart).

**Figure 3.**
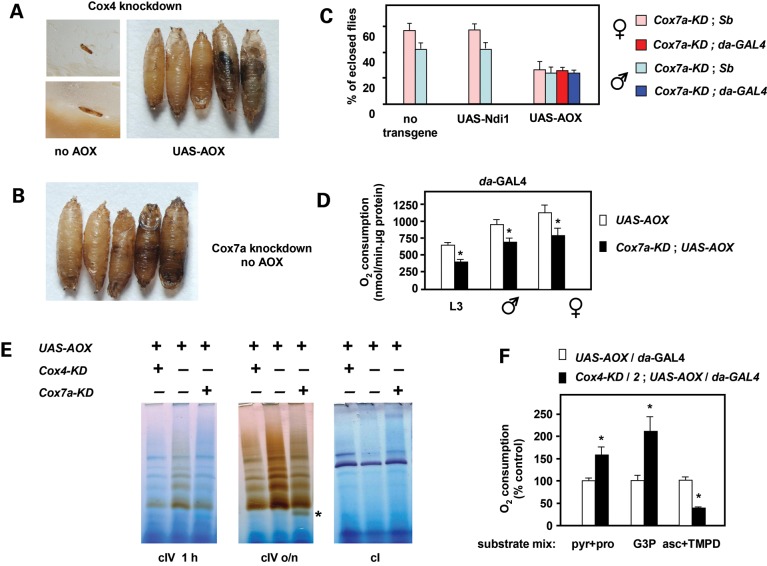
Partial rescue of global COX deficiency by *UAS-AOX*. (**A**) Micrographs illustrating typical phenotypes produced by knockdown of Cox4 (*Drosophila* gene *CoIV*) driven by *da-GAL4*. In the absence of transgenic rescue, progeny arrested as abnormal L1/L2 larvae. Co-expression of *UAS-AOX* enabled development to proceed as far as late pupa, although few flies eclosed. See also Supplementary Material, Figure S3A. (**B**) Micrograph illustrating typical pupal-lethal phenotype produced by knockdown of the major Cox7a-encoding isogene *CG9603*, driven by *da-GAL4*. (**C**) AOX rescue of pupal lethality from Cox7a (*CG9603*) knockdown. Proportion of eclosing progeny for different genotypes as shown, means ± SD from 3 or more biological replicates. Flies with the *da-GAL4* driver have normal bristles, distinguishing them from those with the *Sb* balancer. Expression of UAS-AOX produced a full rescue whereas UAS-Ndi1 expression produced none. (**D**) Ascorbate/TMPD-driven oxygen consumption of homogenates from *UAS-AOX* expressing L3 larvae or adult flies, with or without knockdown of Cox7a (*CG9603*). Means ± SD from 3 or more biological replicates; asterisks indicate significant differences (*P* < 0.01, Student's *t* test, two-tailed, unequal variances). (**E**) BNE gels of mitochondrial extracts (37.5 μg mitochondrial protein per lane) from L3 larvae of the genotypes shown, stained histochemically for cI or cIV activity (cIV activity staining performed for the indicated times). See Ref. 10, Figure 3, for migration of major bands in relation to molecular weight markers on these gels. Asterisk indicates assembly sub-complex S3 (12). (**F**) Oxygen consumption of homogenates from L3 larvae of the indicated genotypes, driven by different substrate mixes (pyruvate + proline, G3P and ascorbate + TMPD, respectively, as described in the Materials and methods section for cI-, G3PDH- and cIV-linked respiration. For clarity, oxygen consumption is expressed as a percentage of corresponding values for control larvae expressing AOX but without RNAi. Means ± SD, ≥3 biological replicates; asterisks indicate significant differences (*P* < 0.01, Student's *t* test, two-tailed, unequal variances).

Using *da-GAL4* to drive simultaneously the high-level expression of UAS-AOX and the knockdown of nuclear-coded COX subunits in the whole developing fly, we delineated the limits of AOX rescue of COX deficiency. Knockdown of the somatic Cox4 isogene produced early larval lethality (Fig. 3A), consistent with the involvement of this subunit in an essential early step in cIV assembly, and its requirement for the production of an enzymatically functional complex. Knockdown larvae survived for at least 15 days, but never developed beyond a morphologically abnormal L1 or L2 stage (Fig. 3A), whereas co-expression of UAS-AOX enabled development to proceed as far as the pupal stage (Fig. 3A), albeit with very few flies eclosing (Supplementary Material, Fig. S3A). Blue native polyacrylamide gel electrophoresis (BNE) in-gel histochemistry confirmed the functional knockdown (≥50% decrease of COX activity) at larval stage L3 in the ‘rescued’ flies (Fig. 3E), as did polarography using a cIV-specific substrate mix (Fig. 3F). Interestingly, oxygen consumption driven by cI- or G3PDH-linked substrates was increased compared with control larvae, suggesting a compensation for missing proton-pumping activity in response to AOX ‘replacement’ of the depleted cIV.

Expression of UAS-Ndi1 as a control produced no rescue of Cox4 knockdown (Supplementary Material, Fig. S3A). Knockdown of Cox5b produced a similar larval-lethal phenotype that was also partially rescued by *UAS-AOX*, allowing flies to develop to pupal stage, with ∼5–10% of pupae eclosing as viable flies (Supplementary Material, Fig. S3B).

Subunit Cox7a is required for the assembly of the fully functional holoenzyme, but not for the formation of an enzymatically active sub-complex (denoted as assembly intermediate S3). Knockdown of the major isogene for Cox7a (CG9603) also resulted in lethality, but in contrast to the early larval lethality produced by knockdown of core subunits, Cox7a knockdown produced lethality only at the pupal stage (Fig. 3B). This was completely rescued by co-expression of UAS-AOX (Fig. 3C), but not by UAS-Ndi1 (Fig. 3C). Consistent with the incorporation of Cox7a at a late stage in cIV assembly and the partial redundancy of the major Cox7a-encoding isogene CG9603 with isogene CG34172 in some tissues, knockdown of CG9603 resulted in only a partial loss of respiratory capacity in the whole fly, based on polarography (Fig. 3D) or BNE in-gel histochemistry (Fig. 3E), with a clear accumulation of assembly sub-complex S3 in L3 larvae.

### AOX-rescuable pupal lethality due to COX knockdown is primarily a muscle phenotype

**Figure 4.**
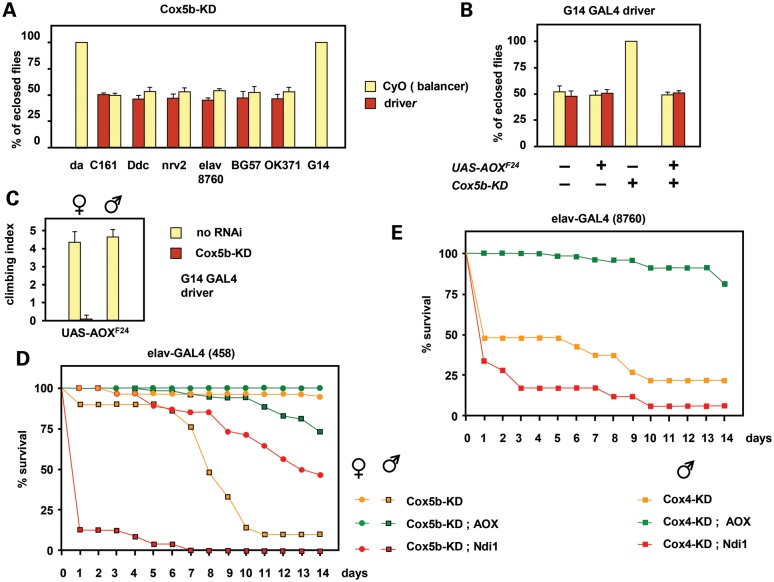
AOX rescue of tissue-restricted cIV knockdown. (**A**–**C**) Knockdown of Cox5b (*Drosophila* gene *CoVb*) using the drivers indicated. Flies with the CyO balancer marker are progeny from the same crosses, but without driver. (A and B) Proportion of eclosing progeny of the genotypes indicated, means ± SD for 3 or more biological replicates. (C) Climbing index (defined as in the Materials and methods section) of 1-day-old UAS-AOX transgenic flies bearing the G14 driver, with and without knockdown of Cox5b. Means ± SD of 10 batches of 5 flies, for each sex and genotype. Note that the AOX-rescued Cox5b-knockdown males were unable to climb at all in this experiment. (**D** and **E**) Survival curves of flies of the indicated genotypes, following Cox5b or Cox4 (*Drosophila* gene *CoIV*) *knockdown* and transgene expression driven by (D) *elav-GAL*4 strain 458 or (E) 8760, as shown. Data are means from two independent experiments. See also Supplementary Material, Figure S4.

To test the tissue(s) in which COX expression is critical for the completion of development, we employed a set of tissue-specific drivers to knockdown either of the enzymatically crucial subunits Cox5b (Fig. 4A) or Cox6b (Supplementary Material, Fig. S4A). No lethality was produced by Cox5b knockdown using any of five nervous system-specific drivers, two of which are active in all neurons (*elav-GAL4* located on chromosome 3, Bloomington strain 8760 and *nrv2-GAL4*), nor were the progeny flies bang-sensitive (Supplementary Material, Fig. S4B). Lethality was also not produced with driver BG57 (Fig. 4A), which expresses in larval muscles from L2 stage and in pupal and adult stages predominantly in abdominal muscle (Supplementary Material, Fig. S5A). However, knockdown using driver G14, which expresses mainly in the thoracic muscles (Supplementary Material, Fig. S5B), produced lethality (Fig. 4A and B), with flies dying at the late pupal stage or during attempted eclosion. Cox6b knockdown gave similar results (Supplementary Material, Fig. S4A). Co-expression of AOX in the same tissues via UAS-AOX rescued the lethality of Cox5b knockdown by the G14 driver (Fig. 4B). Multiple copies of *tub-AOX* also rescued this lethality, though less completely (Supplementary Material, Fig. S4C). Flies rescued by UAS-AOX from the muscle-specific lethality of Cox5b knockdown nevertheless manifested a severe locomotor defect (Fig. 4C), and all died within 2 weeks, as a result of becoming trapped in the food.

### AOX rescue of phenotypes produced by COX knockdown using neuron-specific drivers

The failure to induce lethality by COX knockdown in neurons is superficially surprising. We first verified this for knockdown of a further COX subunit (Cox4), which gave a similar result (Supplementary Material, Fig. S4D). Next, we tested a second *elav-GAL4* driver located on chromosome X (Bloomington strain 458). Different crosses were implemented, so as to study females heterozygous for the driver, plus males hemizygous for the driver, or control males lacking the driver completely (Supplementary Material, Fig. S4E, Supplementary Material, Table S1), as well as any rescue produced by UAS-AOX (or UAS-Ndi1). In general, *elav-GAL4* driver strain 458 produced stronger effects than driver strain 8760, and stronger phenotypes for knockdown of Cox4, which was now almost completely lethal (Supplementary Material, Fig. S4E and F), than for Cox5b. Males were also more affected than heterozygous females. Expression of AOX, but not Ndi1, improved all phenotypes. Viable Cox5b-knockdown flies were transferred to fresh vials and their survival tracked over 2 weeks (Fig. 4D). All genotypes manifested locomotor impairment after several days, which was more severe in males. Females with or without co-expression of AOX survived throughout the experiment, but half of the females co-expressing Ndi1 died by 2 weeks. Cox5b-knockdown males had a mean lifespan of just 8 days, but co-expression of AOX enabled most flies to survive at least 2 weeks. In contrast, Cox5b-knockdown males co-expressing Ndi1 all died after only a few days.

When knockdown was driven by *nrv2*-GAL4 or the autosomally located *elav*-GAL4 driver (Bloomington strain 8760) the outcome of similar survival experiments was much milder. Cox4 knockdown again gave a stronger phenotype than that of Cox5b, and was again stronger in males than females. After 2 weeks, some of the flies with *Cox4* knocked down by the *nrv2*-GAL4 driver had died (though this varied between experiments), whereas *Cox5b* knockdown using the *nrv2*-driver did not impair survival over 2 weeks at all. *Cox4* knockdown by the autosomal elav-GAL4 driver (Fig. 4E) produced a similar effect as *Cox5b* knockdown by the stronger, X-chromosomal *elav*-GAL4 driver (Fig. 4D). Knockdown flies were able to climb, and were observed feeding, although mostly remained motionless, whereas flies co-expressing AOX were normally active and able to fly. Most knockdown males died within a few days, a phenotype exacerbated by co-expression of Ndi1, whereas ∼80% of males co-expressing AOX survived at least 2 weeks. Females were less affected, though followed a similar pattern as with Cox5b knockdown driven by X-chromosomal *elav*-GAL4, with AOX affording protection against early death.

To account for the inconsistency between the effects of knockdown using different neuron-specific drivers we re-examined the tissue-specificity of these drivers, using UAS-GFP (Stinger or mCD8) as a reporter (Supplementary Material, Fig. S5) and by immunocytochemistry and histochemistry (Supplementary Material, Fig. S6). These experiments revealed that expression driven by *nrv2-GAL4* driver was indeed much weaker than that driven by *elav-GAL4*, while the latter gave also a very weak expression in thoracic muscles at pupal stage (see legends to Supplementary Material, Figs S5 and S6 for detailed explanations). Serial sections of the brain and thoracic muscle revealed no gross anatomical defects from Cox4 knockdown using even the strongest *elav*-GAL4 driver (Supplementary Material, Fig. S6G).

### AOX partially rescues adult neurodegeneration caused by COX deficiency

In order to confirm that the degenerative phenotypes produced by *elav*-GAL4 driven COX knockdown and partially rescued by AOX expression were indeed neuronal, we adopted two strategies. First, we took advantage of the fact that Cox7a in muscle is predominantly encoded by isogene CG34172, allowing us to knock down the ubiquitously expressed isogene CG9603 specifically in neurons using *elav*-GAL4, with minimal effects on muscle. Secondly, we analysed the effects of AOX expression in the *levy^1^* mutant in the gene encoding Cox6a, a subunit required for dimerization of cIV. The splice-site mutation results in a frameshift early in the polypeptide sequence, and produces an adult-onset neurodegenerative phenotype (19).

**Figure 5.**
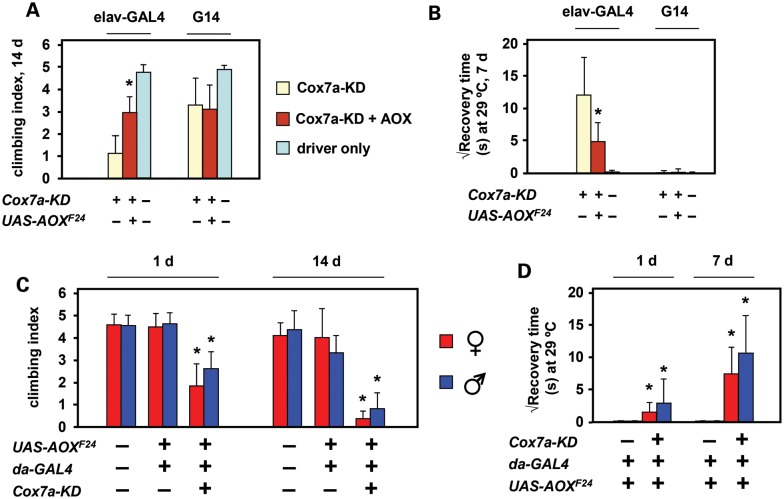
AOX partially rescues neuronal phenotypes resulting from Cox7a knockdown. (**A** and **C**) Climbing index and (**B** and **D**) bang sensitivity at 29°C of flies of the indicated genotypes, ages and sex. Means ± SD generated by analysis of batches of >50 individual flies of each class. Where, indicated, *P* < 0.001, Student's *t* test, two-tailed, unequal variances, (A and B) comparing flies knocked down for Cox7a (isogene *CG9603*) with and without co-expression of AOX or (C and D) comparing flies of a given sex expressing AOX, with or without concomitant knockdown of Cox7a.

Neuronal depletion of Cox7a driven by *elav*-GAL4 resulted in locomotor dysfunction (Fig. 5A) and seizure sensitivity at 29°C (Fig. 5B), both of which were partially rescued by co-expression of AOX. Neither phenotype was produced by muscle-specific knockdown using the G14 driver (Fig. 5A and B), as expected, given that Cox7a expression in muscle relies mainly on isogene CG34172. Cox7a function is also required during development (Fig. 3C), but AOX-rescue of the lethality gave an adult phenotype similar to that seen in flies specifically knocked down for Cox7a and rescued by AOX co-expression in neurons, namely a mild but progressive locomotor defect (Fig. 5C) and seizure sensitivity at 29°C, which was also progressive (Fig. 5D). However, the rescued flies showed normal survival at 2 weeks. When mated to wild-type flies of the opposite sex, males and females were both fertile and their progeny appeared normal. This contrasts with the phenotype exhibited by flies knocked down for subunits of cI, but rescued by Ndi1 (Supplementary Material, Fig. S7), which includes pronounced developmental delay, bang sensitivity at room temperature immediately upon eclosion, and male sterility.

**Figure 6.**
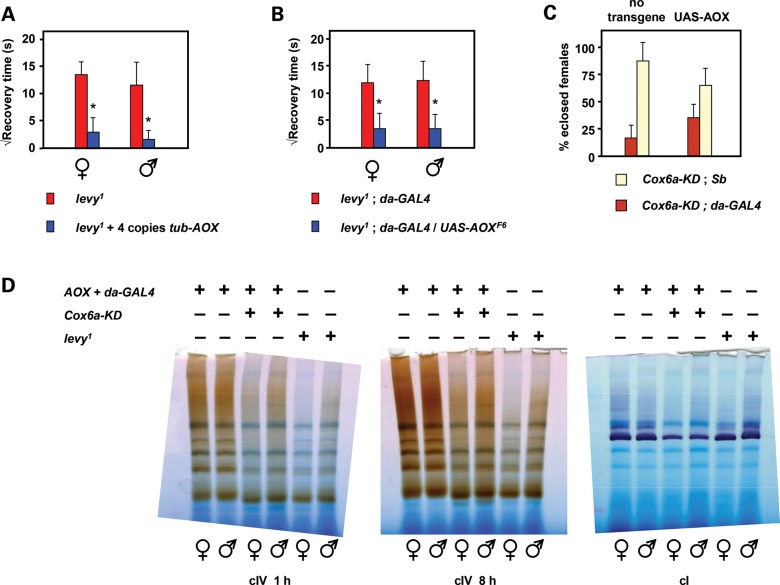
AOX partially rescues Cox6a-dependent neurodegeneration. (**A** and **B**) Rescue of seizure sensitivity of 7-day-old *levy^1^* flies maintained at 29°C. Means ± SD of square root of recovery time from mechanical shock, for batches of 50–100 individual flies of the sex and genotypes indicated. Note that, control flies do not show any such bang sensitivity (∼zero recovery time). (**C**) UAS-AOX rescue of semi-lethality of Cox6a knockdown (*Drosophila* gene *levy*) in female flies. Means ± SD from 3 or more biological replicates. (**D**) BNE gels (as Fig. 3E) from 1-day-old adults of the genotype and sex shown, stained histochemically for cI or cIV activity (cIV activity staining performed for the indicated times). See also Supplementary Material, Figure S7. Where, indicated, *P* < 0.001, Student's *t* test, two-tailed, unequal variances, comparing flies of the given sex, expressing AOX or not. *P* = 0.051 in (C).

Ubiquitous AOX expression using either *tub-AOX* (Fig. 6A) or *UAS-AOX* driven by da-GAL4 (Fig. 6B) also alleviated the temperature-dependent seizure sensitivity exhibited by *levy^1^* mutant flies, although effects on lifespan at 29°C were minimal (Supplementary Material, Fig. S8A–C). Expression of *UAS-Ndi1* in place of *UAS-AOX* did not alleviate the seizure sensitivity of *levy^1^* flies (Supplementary Material, Fig. S8D), instead mildly exacerbated it, similar to the observation that Ndi1 expression enhanced the degenerative effects of *Cox5b* knockdown by *elav*-GAL4 (Fig. 6D and E).

Although *levy^1^* was previously suggested to be a null mutation (of Cox6a), we also tested the effects of Cox6a knockdown by RNAi. Ubiquitous knockdown was semi-lethal to females at pupal stage (Fig. 6C), although the degree of male lethality varied between experiments. Eclosing flies failed to inflate their wings and died in the food shortly after eclosion. Co-expression of UAS-AOX partially rescued female lethality (Fig. 6C, Supplementary Material, Fig. S8E), but the resulting flies were still weak. Knockdown of Cox6a using the muscle-specific driver G14 did not produce the uninflated wings phenotype (0 of 143 flies analysed), whereas *elav-GAL4* did so (223 of 237 flies). Co-expression of AOX rescued this phenotype, with only 9 of 129 flies failing to inflate wings at eclosion (Supplementary Material, Fig. S8E). The Cox6a knockdown phenotype thus appears to be neuronal and again partially rescued by AOX.

The more severe organismal phenotype produced by Cox6a knockdown than by the *levy^1^* splice-site mutation in Cox6a was reflected in a more severe biochemical phenotype as detected by BNE in-gel histochemistry (Fig. 6D). BNE gels revealed a multiplicity of complexes showing COX activity, corresponding to monomeric and dimeric cIV, as well as supercomplexes that were not fully characterized. Knockdown of Cox6a resulted in a clear and reproducible increase in the mobility of the monomeric complex, and a substantial decrease in the abundance of all multimeric and supercomplexes. One of these comigrated on gels with the major complex exhibiting cI activity, and cI activity based on this assay was also clearly decreased in Cox6a knockdown flies, though was hardly affected by the *levy^1^* mutation.

Table 2 shows a summary of the different phenotypes produced by knockdown of COX subunits using various drivers, and their alleviation by *tub*-AOX and/or UAS-AOX in the experiments described above.

**Table 2.** Phenotypes produced by knockdown or mutation of COX subunits, and rescue by AOX

	Genetic lesion^b^	Phenotype	Phenotypic rescue by AOX	Figure
Cox4	Ubiquitous *CoIV* knockdown	Early larval lethal	Pupal lethal with tiny number of eclosed adults	3A, Supplementary Material, Figure S3A
	*CoIV* knockdown in neurons	Pupal semi-lethal (most flies too weak to eclose), surviving adults have short lifespan (<1 week), varies with severity of knockdown	Viable, with locomotor defect but increased lifespan (>2 weeks), varies with severity of knockdown, but always improved	4E, Supplementary Material, Figure S4D–F
Cox5a	Ubiquitous *CoVa* knockdown (partial)	Lethal^c^	Viable, but with 1–2 days developmental delay	2E, Supplementary Material, Figure S2D
Cox5b	Ubiquitous *CoVb* knockdown (partial)	Lethal	Viable, but with 1–2 days developmental delay, adult locomotor defect	2F, Supplementary Material, Figure S2D and F
	Ubiquitous *CoVb* knockdown (severe)	Early larval lethal	Semi-lethal: some viable adults	Supplementary Material, Figure S3B
	*CoVb* knockdown in neurons	Pupal semi-lethal, adult females have short lifespan (∼1 week), varies with severity of knockdown	Viable, with locomotor defect but increased lifespan (>2 weeks), varies with severity of knockdown but always improved	4A and D, Supplementary Material, Figure S4B and E
	*CoVb* knockdown in larval and adult abdominal muscle	Viable^c^	nt^d^	4A
	*CoVb* knockdown in thoracic muscle muscle	Pupal-lethal	Viable, but with locomotor defect	4A–C
Cox6a	*levy^1^* mutant	Adult-onset, progressive neurodegeneration and curtailed lifespan	Greatly alleviated neurodegeneration; no effect on lifespan	6A and B, Supplementary Material, Figure S8A–D
	Ubiquitous *levy* knockdown	Lethal in pupa, or immediately after eclosion (uninflated wings)	Less pupal lethality (in females). viable after eclosion (normal wings)	6C, main text
	*levy* knockdown in neurons	Lethal immediately after eclosion (uninflated wings)	Viable after eclosion (normal wings)	Supplementary Material, Figure S2E
	*levy* knockdown in thoracic muscle	Viable	Viable	Main text
Cox6b	Ubiquitous *CoVIb* knockdown (partial)	Lethal	Viable	Supplementary Material, Figure S2C
	*CoVIb* knockdown in larval and adult abdominal muscle	Viable	nt	Supplementary Material, Figure S4A
	*CoVIb* knockdown in thoracic muscle muscle	Pupal (semi-)lethal	Viable	Supplementary Material, Figure S4A
	*CoVIb* knockdown in neurons	viable	nt	Supplementary Material, Figure S4A
Cox6c	Ubiquitous *cyclope* knockdown (partial)	Semi-lethal, survivors eclose with 2–3 days delay	Viable, <1 day developmental delay	2A and D
Cox7a	ubiquitous *CG9603* knockdown	Pupal lethal	Viable, with mild progressive neurodegeneration	3B and C, Supplementary Material, Figure S5C and D
	*CG9603* knockdown in neurons	Neurodegeneration	Milder neurodegeneration	5A and B
	*CG9603* knockdown in thoracic muscle	Wild-type	Wild-type	5A and B
Surf1 (assembly factor)	*Surf1* knockdown (partial)	(Pupal) semi-lethal	Viable	2B and C

^a^Using mouse nomenclature, as in Table 1.

^b^Official gene symbols/names as in www.flybase.org.

^c^Phenotypes listed only as lethal or viable were not investigated further.

^d^Not tested.

## DISCUSSION

### AOX can partially replace the functions of COX *in vivo*

Despite a much lower level of expression than with the GAL4 system, AOX expressed constitutively under the α-tubulin promoter was effective in combating diverse insults affecting cIV integrity or activity. Flies were protected from cyanide toxicity and from genetic manipulations of Cox6c (*cyclope*) or the cIV assembly factor *Surf1*, to approximately the same extent as when AOX expression was driven by ubiquitously expressed GAL4 (8). These findings indicate, furthermore, that AOX rescue driven by GAL4 or GeneSwitch is not due to promoter dilution effects, and thus validate the use of GAL4 drivers in the remainder of the experiments reported here.

The phenotypes associated with knockdown of most nuclear-coded subunits of cIV were significantly alleviated by *tub-AOX* expression, including Cox4, Cox5a, Cox5b, Cox6a, Cox 6b and Cox6c. *tub-AOX* also partially complemented a null mutant in the single-copy gene for Cox6a (*levy^1^*). Although *tub-AOX* expression of was 3- to 5-fold less in adult females than males (Fig. 1A and B), the phenotypic rescue of COX inhibition that it produced was not substantially different between the sexes, suggesting that only a modest expression of AOX suffices to ameliorate the major consequences of severe COX deficiency.

The complete absence of COX activity, e.g. via a null mutation for Cox6c (*cyclope*), produces early developmental lethality which AOX is unable to compensate (8). However, AOX was able to complement the lethality of a partial loss of COX activity, such as produced by a limited RNAi targeted on essential subunits (Figs 2–4). This allows the level of COX activity which is needed in order to sustain development to be crudely estimated. Thus, the ∼50% drop in the level of COX activity in L3 larvae, brought about by da-GAL4-driven Cox4 knockdown (Fig. 3E and F), or Cox5b knockdown by the GeneSwitch driver in presence of 0.5 μm RU486 (Supplementary Material, Fig. S2B), is larval lethal in the absence of AOX, and very close to the amount of knockdown that blocks the completion of metamorphosis even in the presence of AOX (Supplementary Material, Fig. S3A). Note that many more flies completed development when Cox5b knockdown was brought about by GeneSwitch, with AOX supplied from just two copies of *tub-AOX* (Fig. 2F), than when knockdown and rescue were both driven by *da-GAL4* (Supplementary Material, Fig. S3B). This may be explained by the fact that the GeneSwitch driver is dependent on the supply of the inducing drug, so that during metamorphosis, when the flies are not eating, but the drug previously ingested can be metabolized, GeneSwitch driven-RNAi decays.

*da*-GAL4-driven RNAi targeted against genes for catalytically essential subunits, such as Cox4, Cox5a or Cox5b, was lethal at an early (L1/L2) larval stage, although the abnormal larvae survived a further 2 weeks without further growth. Similar phenotypes have been seen for other manipulations affecting mitochondrial OXPHOS, such as knockdown of mitochondrial transcription factor mtTFB2 (43) or the cIV assembly factor CCDC56 (44). The implication is that larvae can survive for long periods on the small amount of OXPHOS capacity acquired maternally, but require additional respiratory or ATP-generating potential for growth and developmental progression. AOX expression allowed this developmental block to be overcome, although ‘rescued’ flies still died during metamorphosis if COX activity was below a critical level, and the few flies that eclosed were typically weak and short-lived. The ability of AOX to rescue early larval but not pupal lethality does not mean that the requirement for OXPHOS is greater during metamorphosis than during larval growth: the maternal contribution to total COX activity, which may be assumed to be significant in larvae, becomes enormously diluted during development, on a per cell basis.

### COX deficiency produces a range of adult phenotypes in *Drosophila*

Surprisingly, the knockdown of catalytically essential subunits by pan-neuronal drivers, as well as drivers active in various different subsets of neurons, was non-lethal even in the absence of AOX (Fig. 4). Previous data are consistent with the idea that neurons can develop and survive long periods with profound OXPHOS deficiency. Mice with only 30% of the wild-type COX activity in the brain, due to a deletion of the *Surf1* gene, are nevertheless viable (45), and postnatal deletion of the *Tfam* gene in mouse forebrain neurons produces only a late-onset neurodegeneration, which manifests pathologically a full month after profound neuronal COX deficiency is seen histochemically (46). Chimeric mice created using this model also show long-term survival, even if >50% of neurons are respiration-deficient (47). In *Drosophila*, even severe neurodegenerative phenotypes associated with mitochondrial dysfunction, are non-lethal during development (48,49).

In contrast, muscle-specific knockdown of COX during development, using GAL4 driver G14, was pupal-lethal in the absence of AOX. This lethality was not produced by driver BG57, which is active in larval and adult abdominal muscles, but not the thoracic muscles that are targeted by G14 (Supplementary Material, Fig. S5A and B), indicating the latter as the critical target. The lethality of Cox5b knockdown driven by G14 was fully rescued by co-expression of AOX, although when knockdown was driven by *da*-GAL4 the result was still almost complete pupal lethality, suggesting that some COX activity in tissues other than muscle is needed for the completion of development. Moreover, while the developmental block caused by muscle-specific knockdown of Cox5b was rescued by AOX, the eclosed flies were very weak. AOX can thus complement COX deficiency sufficiently to enable muscle development but can only do so incompletely for muscle function, indicating that at least some residual proton-pumping activity at cIII/cIV is essential for full activity of the tissue. This may mirror the prominence of muscle involvement in those human mitochondrial diseases where the primary defect causes only a decrease in the activity of cIV (20) or cIII (50).

The different phenotypes produced by cIV knockdown using pan-neuronal drivers partly reflect their different strengths, as revealed by GFP expression (Supplementary Material, Fig. S5). Thus, the degenerative phenotypes produced by Cox4 knockdown range from semi-lethality with the stronger, X-chromosomal *elav*-GAL4 driver (strain 458), to no detectable impairment within the first 2 weeks of adult life, for *nrv2*-GAL4. Autosomal *elav*-GAL4 (strain 8760), which drives GFP expression less strongly in the adult and pupa (Supplementary Material, Fig. S5F–I), gave an intermediate phenotype. The stronger *elav*-GAL4-driven phenotype in males is an expected consequence of dosage compensation of an X chromosome-derived promoter. In addition, based on GFP (Supplementary Material, Fig. S5F–I) and AOX expression (Supplementary Material, Fig. S6B), as well as cIV knockdown as judged by immunocytochemistry (Supplementary Material, Fig. S6D) and histochemical staining (Supplementary Material, Fig. S6E and F), the *elav*-GAL4 drivers are weakly active also in developing thoracic muscle from the pupal stage. This complicates the interpretation of the organismal phenotypes produced using these drivers. The weakness, inactivity and locomotor dysfunction produced by *elav*-GAL4-driven Cox4, Cox5b or Cox6a knockdown are similar to the effects produced by muscle-specific driver G14, though vary in degree. They appear to constitute a stable, developmentally determined phenotype distinct from the progressive neurodegeneration exhibited by the Cox6a mutant *levy^1^*, which also resembles the *other*, more progressive effects of cIV knockdown produced by the *elav*-GAL4 drivers.

However, the effects of knockdown of the major somatic isoform of Cox7a (Fig. 5) indicate that the *elav*-GAL4-driven phenotypes produced by knockdown of core subunits of cIV are, in fact, predominantly or exclusively neural. Data from flyatlas.org indicate that the isogene *CG9603* accounts for >99% of Cox7a expression in the brain. However, in adult carcass, which consists mainly of muscle tissue, the expression of Cox7a isogene *CG34172* predominates. Consistent with this, the phenotypes produced by global knockdown of isogene *CG9603* using *da*-GAL4 in the presence of UAS-AOX (progressive bang sensitivity and locomotor impairment, Fig. 5C and D) were not seen when using the muscle-specific driver G14 (Fig. 5A and B), but were produced by the *elav*-GAL4 driver (Fig. 5A and B), confirming that these are the characteristic neuronal phenotypes of adult COX deficiency in *Drosophila*. Importantly, these phenotypes were alleviated by the concomitant expression of AOX, even at relatively modest levels, e.g. *tub-AOX* rescue of the *levy^1^* phenotype (Fig. 6A).

### Neuronal COX-deficient phenotypes and bioenergetic insufficiency

AOX rescue of the neuronal phenotypes produced by knockdown of different cIV subunits was not complete. For example, even when AOX was co-expressed, Cox7a knockdown still produced a mild, residual bang sensitivity and locomotor dysfunction that were not seen in wild-type flies, and which worsened with age (Fig. 5C and D). Furthermore, although the temperature-dependent bang sensitivity of *levy^1^* flies was greatly alleviated by AOX, it was not completely abolished (Fig. 6A and B), and the short lifespan of *levy^1^* flies was hardly affected (Supplementary Material, Fig. S7A–C). Importantly, the degree of rescue of the *levy^1^* phenotype was the same (Fig. 6A and B), even when AOX was expressed at a much higher level, driven by *da*-GAL4 (Fig. 1A).

Because it is non-proton-pumping, AOX cannot rescue ATP production at cIII/cIV. However, by releasing electron flow from a block at the level of cIV, AOX expression can restore ATP production due to proton-pumping at cI. In addition, it can decrease other consequences of cIV inhibition, such as excess ROS production (6–9), metabolic acidosis or disturbances to intermediary metabolism and, for unexplained reasons, decreases mitochondrial ROS production even under non-inhibited conditions [(51), Fig. 3]. The question thus arises as to which of these effects underlies the neurodegeneration produced by COX deficiency, even in the presence of AOX. Importantly, in this regard, co-expression of Ndi1 in place of AOX actually worsened the *levy*^1^ phenotype (though this was only statistically significant in males), and also worsened the degenerative effects of Cox4 or Cox5b knockdown produced by the *elav*-GAL4 drivers. Unlike AOX, expressed Ndi1 appears to be constitutively active (10), thus diverting a proportion of the electron flow that should normally pass through cI and decreasing the total amount of proton-pumping for a given respiratory flux. Under conditions where electron flow through cIV is too low, Ndi1 expression should diminish further an already insufficient rate of ATP production. Conversely, it should have no effect on excess ROS production at cIII, since coenzyme Q (CoQ) remains maximally reduced under these conditions. ROS production at cI might actually be mildly alleviated. Thus, our results strongly suggest that the primary mechanism of neurodegeneration caused by COX deficiency is a bioenergetic deficit, and not an effect of excess ROS or metabolic disturbance.

The failure of AOX to improve the short lifespan of *levy^1^* flies, combined with our analysis of the *levy^1^* and Cox6a-KD phenotypes by BNE (Fig. 6D), suggests that the *levy^1^* mutation may also act via another mechanism. The Cox6a gene product appears to be required not only for the dimerization/multimerization of cIV but also the participation of cIV in respiratory supercomplexes, including those that also contain cI (Fig. 6D). Supercomplex formation has elsewhere been shown to be crucial for full respiratory function (18,52). Moreover, the absence of Cox6a results in a functional deficiency of both complexes IV and I. Since other data strongly indicate that loss of cI activity (10,53), especially in the nervous system (54), is a primary cause of ageing, the short lifespan of *levy^1^* flies may be due to cI deficiency and/or loss of cI-containing supercomplexes, rather than COX deficiency as such.

### Loss of proton-pumping at cIV or cI produces distinct organismal phenotypes

The residual phenotypes seen when cIV is functionally replaced by AOX or when cI is functionally replaced by Ndi1 are distinct. AOX-rescued cIV-knockdown flies manifest the developmentally determined muscle weakness and neurodegeneration phenotypes described above, but are fertile, show no bang sensitivity at room temperature when tested 1–2 days after eclosion, and suffer only a short (1–2 days) developmental delay. In contrast, Ndi1-rescued cI-knockdown flies show a pronounced (3–5 days) developmental delay, bang sensitivity detectable at room temperature immediately after eclosion, and male sterility associated with greatly decreased amounts of sperm, which are also immotile. However, the Ndi1 rescued flies are normally active, indicating unimpaired muscle function, and do not show short lifespan or other signs of neurodegeneration.

These different phenotypes may be considered as an example of the different respiratory thresholds of different tissues (42). COX has previously been suggested to have a low reserve capacity in skeletal muscle, based on biochemical threshold measurements of the different OXPHOS complexes in various rat tissues (42). The dependence of individual tissues on the different OXPHOS complexes is also greatly influenced by the metabolic fuels that are supplied physiologically. For example, inhibition of cI should be much less important in a tissue dependent on oxidation of succinate or α-glycerophosphate than one where pyruvate is the main substrate. In *Drosophila* flight muscle, ATP production depends on a combination of glycolysis and α-glycerophosphate oxidation in mitochondria, which donates directly electrons to cIII via CoQ, using an flavin adenine dinucleotide-linked enzyme GPD2 (55). This α-glycerophosphate cycle also sustains the regeneration of nicotinamide adenine dinucleotide + without production of lactate, and its genetic ablation causes loss of viability (56). This is consistent with the greater dependence of muscle on the activity of cIV as opposed to cI implied by our results, and may also account for the absence of muscle weakness and pupal lethality in *levy^1^* flies, where a major effect of the mutation appears to be the absence of supercomplexes containing cI.

The phenotype of pronounced developmental delay and bang sensitivity is characteristic of *Drosophila* mutants lacking mitochondrial OXPHOS capacity, notably *sesB^1^* (57) and *tko^25t^* (58). cI is the most affected of the OXPHOS complexes in *tko^25t^* (58). Based on the patterns of tissue-specific rescue with the wild-type *tko* gene, bang sensitivity was suggested to be a neural phenotype (59,60). Although *tko^25t^* exhibits a male courtship defect (58) it is not sterile. However, the combined phenotype of bang sensitivity and male sterility has been previously reported in engineered *Drosophila* hypomorphs for porin (61,62), showing a similar sperm defect as seen in Ndi1-rescued cI-knockdown flies. Male infertility comprising low sperm number (oligozoospermia), with loss of sperm motility (asthenozoospermia) is a common phenotype in humans, and has been reported, in specific populations, in individuals polymorphic for the CAG repeat at the *POLG1* (mtDNA polymerase) locus (63,64), or deriving from a specific mitochondrial haplogroup (65). Since Ndi1 should compensate the redox disturbance and excess ROS production resulting from cI deficiency, our findings indicate a specific importance of proton-pumping at cI for normal sperm differentiation and motility, and may reflect a unique metabolic signature of sperm (66).

### AOX as therapy for COX deficiency based on the *Drosophila* model

Previous studies in *Drosophila* (8,49) indicated that AOX expression is a potentially useful tool for correcting a wide spectrum of genetic defects affecting the mitochondrial cytochrome chain. The present study extends these findings by showing that AOX can overcome both lethal and tissue-specific effects of various types of COX deficiency, notably those affecting early development, muscle differentiation and function and the functional maintenance of the nervous system during adult life (Table 2). AOX was able to rescue phenotypes arising from genetic lesions affecting COX subunits required for early [Cox4, Cox5a (67)], intermediate (Cox5b, Cox6b) and late steps (Cox7a) in cIV assembly, a key subunit (Cox6a) required for cIV multimerization and supercomplex formation, and the assembly factor *Surf1*. COX deficiency in humans also has a diversity of genetic causes, including catalytic (29–31) and structural subunits (27,28), assembly factors (22,23,25,26) and genes required for biosynthesis (32,33). Pathological COX deficiency is ALSO among the most severe manifestations of mitochondrial disease. Developing AOX as a wide-spectrum genetic therapy is thus an attractive, if distant goal.

## MATERIALS AND METHODS

### *Drosophila* stocks and maintenance

*w^1118^* flies, standard balancers, UAS-GFP lines (Stinger with an insertion on chromosome 2 and mCD8 on chromosome 3) and GAL4 driver lines were obtained from stock centres. G14-GAL4 (68) was a kind gift from Professor John C. Sparrow (University of York) and the *tubulin*-GeneSwitch (*tub-GS*) driver (69) from Dr Scott Pletcher (University of Michigan). The *Surf1-KD* line 79.1 (70), *levy*^1^ mutant strain (19), UAS-AOX transgenic lines AOX^F6^ and AOX^F24^ (8) and UAS-Ndi1 transgenic line A46 (10) were described previously. RNAi stocks targeted against *cyclope*, *CoIV*, *CoVa*, *CoVb*, *levy*, *CoVIb*, *CG9603*, *CG6020* and *CG3683* (71) were from the Vienna Drosophila RNAi Centre (VDRC). All flies were maintained in standard medium (8), with addition of RU486 (Mifepristone, Sigma, St Louis, MO, USA) for induction of expression using the *tub*-GS driver.

### Generation of tub-AOX transgenic lines

Trasgenic lines containing *Ciona intestinalis AOX* under the control of the *Drosophila αTub84B* promoter were created, using, a modified pGREEN-H-Pelican vector (72) (*Drosophila* Genomics Resource Center, Bloomington, IN, USA) as previously described (8). Insertion sites were determined by inverse polymerase chain reaction (PCR) (8).

### Developmental time, behavioural, toxin resistance and lifespan assays

Crosses were conducted in triplicate, and mean developmental time to eclosion at 25°C and bang sensitivity measured as described previously (73). Resistance to cyanide and climbing ability were assayed essentially as described in Ref. (8), with minor modifications (8, see SI). For lifespan curves, virgin females and males were collected in sets of 10 flies per vial, and transferred to fresh food vials three times a week, as previously (10). At least 10 vials of each sex and genotype were used in life-span experiments, or 3 vials of each sex and genotype when measuring survival over just 2 weeks.

### Protein analysis

Mitochondria from batches of 80–100 adult flies or pupae were isolated essentially as described (74). For isolation of mitochondria from L3 larvae, 0.01 m freshly neutralized cysteine hydrochloride was added to the isolation buffer. SDS-PAGE and western blotting using antibodies against AOX and ATP synthase subunit α were as described previously (8). BNE and in-gel activity staining of mitochondrial enzymes (54) used NativePAGE Novex Bis-Tris Gel System (Invitrogen Life Technologies, Carlsbad, CA, USA) and batches of 100 µg of isolated mitochondria.

### RNA extraction and qRT-PCR

RNA extraction from *Drosophila* adults and larvae, cDNA synthesis, quantitative real time-polymerase chain reaction (qRT-PCR) and data analysis were performed as described for *Surf1* mRNA in Ref. (8), using primer sets shown in Supplementary Material, Figure SI.

### Metabolic analyses

Polarography was performed using a high-resolution Oroboros 2K respirometer for whole homogenates, as described previously (75). COX activity was measured using the CYTOCOX1 kit (Sigma), according to manufacturer's recommendations.

### Imaging

Imaging of the head and thorax was carried out by haematoxylin and eosin staining of paraffin sections. For immunocytochemistry, heat- and Triton X-100-treated paraffin sections were incubated with primary antibodies against COXIV (Abcam, rabbit), ATP5A (Abcam, mouse) or AOX (21st Century Biologicals, rabbit), with appropriate secondary antibodies (Invitrogen). Slides were imaged by spinning disc confocal microscopy. Staining for COX and SDH activity was carried out on cryosections, essentially as described previously described (76, see Supplementary Material, Fig. SI).

GFP expression patterns generated by various GAL4 drivers were determined by crossing homozygous driver and GFP reporter lines, with imaging of larvae and dissected organs by fluorescence microscopy. No image manipulation was done, other than standard brightness and contrast optimization.

For further details see SI.

## SUPPLEMENTARY MATERIAL

Supplementary Material is available at *HMG* online.

## FUNDING

This work was supported by funding from the Academy of Finland Tampere University Hospital Medical Research Fund; the Sigrid Juselius Foundation and European Research Council (advanced grant 232738 to H.T.J., starting grant 260632 to A.S.). P.R. received support from AMMi (Association contre les Maladies Mitochondriales), from Action Rémy and ANR projects MitOxy and AifInter. Funding to pay the Open Access publication charges for this article was provided by University of Tampere.

## Supplementary Material

Supplementary Data
