# Supplementary material for: Expression of alternative oxidase in Drosophila ameliorates diverse phenotypes due to cytochrome oxidase deficiency
Source: Hum Mol Genet. 2013 Nov 29;23(8):2078–93. doi: 10.1093/hmg/ddt601 (PMC3959817; doi:10.1093/hmg/ddt601)
Supplement: Supplementary Data [file supp_23_8_2078__index.html]

Expression of alternative oxidase in Drosophila ameliorates diverse phenotypes due to cytochrome oxidase deficiency — Expression of alternative oxidase in Drosophila ameliorates diverse phenotypes due to cytochrome oxidase deficiency — Supplementary Data 

# Expression of alternative oxidase in *Drosophila* ameliorates diverse phenotypes due to cytochrome oxidase deficiency

## Supplementary Data

Supplementary Data

**Files in this Data Supplement:**

- Supplementary Data - Pdf file
