# Supplementary material for: Expression of alternative oxidase in Drosophila ameliorates diverse phenotypes due to cytochrome oxidase deficiency
Source: Hum Mol Genet. 2013 Nov 29;23(8):2078–93. doi: 10.1093/hmg/ddt601 (PMC3959817; doi:10.1093/hmg/ddt601)
Supplement: Supplementary Data [file supp_ddt601_ddt601supp.pdf]

Expression of alternative oxidase in *Drosophila* ameliorates diverse phenotypes due to cytochrome oxidase deficiency

Kia K. Kemppainen, Juho Rinne, Ashwin Sriram, Matti Lakanmaa, Akbar Zeb, Tea Tuomela, Anna Popplestone, Satpal Singh, Alberto Sanz, Pierre Rustin & Howard T. Jacobs

SUPPLEMENTARY INFORMATION

SUPPLEMENTARY MATERIALS AND METHODS

Drosophila stocks and maintenance

w^{1118} flies, standard balancers, UAS-GFP lines (Stinger with an insertion on chromosome 2 and mCD8 on chromosome 3) and GAL4 driver lines (Bloomington strain numbers shown in parentheses): *da*-GAL4 (8641), BG57 (32556), *elav*-GAL4 (8760, with an insertion on chromosome 3; 458 on the X-chromosome), *nrv*2-GAL4 (6800), C161 (27893), Ddc (7010), and OK371 (26160) were obtained from stock centres. G14-GAL4 (68) was a kind gift from Prof. John C. Sparrow (University of York) and the *tubulin*-GeneSwitch (*tub-GS*) driver (69) from Dr Scott Pletcher (Baylor College of Medicine). The *Surf1-KD* line 79.1 (70), $levy^1$ mutant strain (19), UAS-AOX transgenic lines AOX^{F6} and AOX^{F24} (8) and UAS-Ndi1 transgenic line A46 (10) have been described previously. RNAi stocks from the Vienna Drosophila RNAi Centre (VDRC) were as follows (gene names in parentheses): 13403 (*CG14028, cyclope)*, 109338 (*CG10664, CoIV*), 44490 (*CG14724, CoVa*), 30892 (*CG11015, CoVb*), 101523 (*CG17280, levy*), 26848 (*CG14235, CoVIb*), 106661 (*CG9603*), 13131 (*CG6020*) and 46797 (*CG 3683*) (71). All flies were maintained in standard medium as described in supplementary data of (8). For induction of expression using the *tub*-GS driver, flies were cultured in the presence of various doses of RU486 (Mifepristone, Sigma).

Generation of tub-AOX transgenic lines

The *Drosophila* promoter for the Ŭ-tubulin (*αTub84B*) gene and the coding region of the *Ciona intestinalis AOX* gene were joined together by chimeric PCR, using fly genomic DNA and the pUAS-AOX-H-Pelican construct (8) as respective templates, and primers with complementary 14 bp overhangs. This chimeric product was cloned using Zero Blunt TOPO PCR Cloning Kit (Invitrogen) under manufacturerӧs recommended conditions, then excised and recloned into a modified pGREEN-H-Pelican vector (8, 72) (*Drosophila* Genomics Resource Center, Bloomington, IN, USA). All restriction digestions were carried out under manufacturerӧs recommended conditions (NEB, Fermentas). The construct was restriction-

mapped and sequenced to verify the construction and the absence of mutations. Following microinjection into w^{1118} embryos (VANEDIS Drosophila Injection Service, Oslo, Norway), transgenic progeny were established as independent lines in the w^{1118} background. Insertion sites were determined by inverse PCR as described (8). Lines *tub*-AOX^{35} (intergenic insertion on X-chromosome), *tub*-AOX^{50} and *tub*-AOX^{112} (both on chromosome 2), and *tub*-AOX^{7} (chromosome 3) were retained for further study, maintained as homozygotes, and used to generate lines with multiple insertions on different chromosomes where needed for specific experiments. For further details on the insertions see Figure S1A-D.

Experimental crosses and developmental time

Crosses were conducted in triplicate, typically using 12 virgin females and 6 males, mated for 3 days before tipping to fresh vials for a further 2 days. Mean developmental time to eclosion at 25 °C was measured as described in (73). Tests of the effects of different RNAi constructs using various drivers were generally conducted using parental flies with balancer markers, except where behavioural tests were to be conducted, in which case appropriate parental flies were themselves constructed from balanced stocks, in order to avoid possible confounding effects of the balancers.

Behavioral, toxin resistance and lifespan assays

Bang-sensitivity was measured as described previously (73). For studying temperature-dependent bang-sensitivity, flies were maintained at 29 °C after eclosion, for the times indicated in figure legends. Climbing ability was assayed essentially as described in (8), with minor modifications: virgin females and males were separated into groups of 5 flies in test vials with a line marked at 6 cm. After tapping the flies to the bottom of the vial, the number of flies reaching the line in 10 s was recorded. This was repeated 3 times for each group of 5 flies, giving an average score between 0-5 (the climbing index). Ten sets of flies of each genotype and sex were tested in each experiment. Resistance to cyanide was tested as

described previously (8). Groups of 80-100 flies of each indicated genotype were tested in batches of 10 flies per vial, and the time to complete paralysis of all 10 flies was recorded. For lifespan curves, virgin females and males were collected in sets of 10 flies per vial, and maintained at 25 °C (or 29 °C for *levy*^{1} and *levy*-KD) in standard medium. Flies were transferred to fresh food vials three times a week, counting the number of dead flies with each transfer. At least 10 vials of each sex and genotype were used in life-span experiments. Short-term survival assays were conducted similarly, but using only 3 vials of each sex and genotype.

Protein analysis

Mitochondria from adult flies and pupae were isolated as described (74), from batches of 80-100 flies ground in. 0.5 ml of isolation medium, with final resuspension in 50 εl of isolation medium without BSA. For isolation of mitochondria from L3 larvae, 0.01 M freshly neutralized cysteine hydrochloride was added to the isolation buffer. Protein concentrations were measured using the Bradford assay. SDS-PAGE and Western blotting using antibodies against AOX, ATP synthase subunit Ŭ, NDUFS3 and GAPDH were as described previously (8, 10). Blue native polyacrylamide gel electrophoresis (BNE) and in-gel activity staining of mitochondrial enzymes were performed essentially as described in (54), using NativePAGE Novex Bis-Tris Gel System (Invitrogen) and batches of 100 µg of isolated mitochondria. Staining for cI activity was for 20 min, for cIV activity for the times indicated in figure legends.

RNA extraction and Q-RT-PCR

RNA from *Drosophila* adults and larvae was extracted as described (8). cDNA synthesis, qRT-PCR and data analysis were performed as described for *Surf1* mRNA in (8), using the following primer sets (all denoted 5´ to 3´): AOX S and AOX R for *AOX* (8), RpL32 F short and RpL32 A Short for *RpL32* (8), Surf1 F and Surf1 R for *Surf1* (70), cypeF: CGCCTACGCCGACTTCTACT and cypeR: GAAGCGCAACTCTTGTTCTACA for

cyclope, CoVa F: CGATGCCTAAGCGGAGAACA and CoVa R: CATCCGCACCATACATTGGA for *CoVa* and 30892 F: CAATGGCATCGATCTGTGGA and 30892 R: ATACCAGTGGCGTGCTCCAA for *CoVb*.

Metabolic analyses

Cytochrome *c* oxidase activity was measured using the CYTOCOX1 kit (Sigma), according to manufacturerôs recommended conditions. Polarography was performed using a high-resolution Oroboros 2 K respirometer for whole-fly homogenates, as described previously (75), sequentially using the following substrate mixes (Sigma): for cI-driven substrate oxidation, 5 mM each sodium pyruvate and proline, plus 1 mM ADP; for G3PDH-driven substrate oxidation: 0.5 εM rotenone followed by 20 mM glycerol-3-phosphate; for cIV-driven substrate oxidation: 2.5 εM antimycin followed by 2 mM ascorbate plus 400 εM TMPD,

Imaging

Imaging of the head and thorax was carried out on flies from which abdomen and wings had been removed prior to fixation in 4% formaldehyde for 3-5 h and paraffin embedding (Sakura Tissue-Tek VIP 4). 4 εm sections (Microm HM310 rotation microtome) were attached to Thermo Scientific SUPERFROST Plus microscope slides at 37 °C overnight. Paraffin was removed using three xylene washes, followed by a descending ethanol series. Sections were analysed both by immunocytochemistry and by haematoxylin and eosin (H&E) to distinguish morphological differences in the brain. For H&E staining slides were first incubated in haematoxylin (Mayers, Merck, diluted 1:4 with distilled water) for 3 min, washed with running tap water for 10 min, then counter-stained in 0.1 % Eosin Y (Histola, with glacial acetic acid added) for 40s. Excess colour was rinsed off with distilled water and slides. Stained sections were dehydrated in an ascending alcohol series, and cleared with xylene before mounting with organic mounting medium (EUKITT) and viewing with an Olympus BX 51 microscope and Olympus ColorView IIIu camera operated by Olympus Cell^{B} software v. 2.4.

For immunocytochemistry, slides were rinsed with TBS-Tween (TBST) before incubation for 30 min at 95 °C in 10 mM citrate buffer, 2.5 mM EDTA, 0.05% Tween 20, pH 6.2. The solution was cooled on ice to room temperature, after which slides were rinsed with TBST, incubated in 0.3 % Triton X-100 in TBST for 30 min, and washed 3 times for 5 min in TBST, all at room temperature. After blocking with 5 % bovine serum albumin (BSA) in TBST overnight at 4° C, primary antibodies were added in TBST containing 5 % BSA, and incubated for 1 h at room temperature. Primary antibodies were against COXIV (Abcam, ab16056, rabbit polyclonal, 1:200), ATP5A (Abcam, ab14748, mouse monoclonal, 1:1000) or AOX (21st Century Biologicals, custom-made rabbit polyclonal PI047AB, 1:1000). Following 3 further 10 min washes in TBST, secondary antibodies (Invitrogen Goat-anti-rabbit Alexa fluor 568, A-11011 or Goat-anti-mouse Alexa fluor 488, A-10680, both at 1:1000 in TBST plus 5% BSA) were added for a further 1 h incubation at room temperature. Slides were washed again with TBST (3 times, 10 min) and a glass coverslip was mounted with ProLong Gold with DAPI (Invitrogen). Slides were imaged using a Spinning Disc Confocal microscope (Nikon Eclipse Ti, Wallac-Perkin Elmer Ultraview system), Andor EMCCD camera and iQ software.

COX and SDH staining (76) was carried out on cryosections, prepared by casting flies anaesthetized with CO_2 into Tissue-Tek O.C.T. mount (Sakura) and snap freezing in isopentane surrounded by liquid N_2. Cryoblocks were cut into 5 µm sections with a Leica CM3050S cryo-microtome at -16-18 °C) and attached to Polylysine slides (Thermo Scientific), which were stored at -20°C then thawed for staining at room temperature. COX activity was visualized by incubating slides for 15 min in 9 ml of 0.05 M phosphate buffer pH 7.4, containing 5 mg 3,3´diaminobenzidine, 20 mg catalase, 10 mg cytochrome c and 750 mg of sucrose, followed by three washes with distilled water. SDH activity was visualized by incubating slides either prestained or not for COX, at 37 °C for 5 min in 10 ml 0.05 M phosphate buffer pH 7.4 containing 10 mg nitroblue tetrazolium and 540 mg sodium

succinate, followed by three washes with distilled water. Stained sections were dehydrated in an ascending alcohol series, and cleared with xylene slides, before mounting and visualization as described above for H&E-staining.

GFP expression patterns generated by various GAL4 drivers were determined by crossing homozygous driver and GFP reporter lines. Flies were left to mate in the dark for 1 d in mating chambers, in order to collect L1, L2 and L3 larvae. Larvae were rinsed in PBS, dried on tissue, then placed on microscope slides cooled to 4 ºC (Thermo-Scientific, Superfrost plus). A drop of 70 % glycerol was added, and larvae were frozen at -20°C 10 (L1) or 20 min (L2/L3). Whole larvae on frozen slides were imaged with an Olympus SZX16 microscope and ColorView IIIu camera, controlled by Olympus Cell^{B} software (v. 2.4), under u.v. light (fluorescent lamp Olympus U-RFL-T). L3 larvae were dissected in PBS using Dumont #55 INOX forceps, on microscope slides with a recessed well and individual organs and tissues were removed for separate imaging. Pupal dissections were performed between stages 8-10, when there was visible eye pigment. Pupae were immobilized on a microscope slide with double-sided tape and the pupal case was gently removed using dissection forceps, beginning from the anterior and lifting the case away from the dorsal side, after which 70% glycerol was added and the whole pupa imaged. Pupae were dissected in PBS to isolate thoracic muscle and the CNS (brain and ventral nerve cord) for imaging. Adult flies were anaesthetized for 10-20 min in otherwise empty vials containing a cotton swab dipped in FlyNap (Carolina Biological Supply Company) Whole flies were imaged under fluorescent and bright light and then dissected. Before dissection flies were dipped into 96 % EtOH for 10 s to soften the cuticle for easier dissection, which was done in PBS, to isolate thoracic muscle, CNS and abdominal organs for individual imaging. Dissection of testes for imaging by phase-contrast microscopy was conducted on 2 day-old males anaesthetized on ice. For video imaging of sperm motility, dissected testes were punctured in PBS containing 1% BSA.

No image manipulation was done, other than standard brightness and contrast optimization.

SUPPLEMENTARY FIGURE LEGENDS

Figure S1

Insertion sites and functionality of *tub-AOX* transgenes

(A-D) Insertion sites and transcriptional orientations of the various transgenes, based on inverse PCR, are shown as red arrows, against GBrowse images of the relevant regions of the genome, downloaded from Flybase. (E) Representative oxygraph traces of isolated mitochondria from adult females of the different (homozygous) transgenic lines, incubated with cI-linked substrate mix, plus the indicated substrates and inhibitors. The relative slopes of the traces before and after cyanide addition, as shown, indicate the proportionate degree of cyanide resistance conferred *in vitro* by the AOX transgene in the three lines, following the same order (tub-AOX^{35} > tubAOX^{7} > tub-AOX^{50}) as *in vivo* cyanide resistance (Fig. 1C) and amount of expression at RNA level following eclosion (Fig. 1A).

Figure S2

Supplementary data on phenotypes and *tub-AOX* rescue of cIV subunit knockdown

(A) Verification of (partial) knockdown at mRNA level for the indicated genes and concentrations of RU486, in presence of the *tub-GS* driver. Means ± SD of Q-RT-PCR measurements on ≥ 3 biological replicates from L3 larvae, normalized to the mRNA levels for each given gene (Cox6c ï *cyclope,* Cox5a ï *CoVa,* Cox5b ï *CoVb*) in the control larvae
A1 (driver plus drug only). (B) COX activity of extracts from L3 larvae knocked down for Cox5b by 0.5 εM RU486, in presence of the *tub-GS* driver, and rescued by 2 copies of *tub*-AOX, alongside value for control larvae (2 copies of *tub-AOX* plus driver and drug, but no RNAi). Means ± SD from ≥ 3 biological replicates, $p < 0.05$ (*) (Studentôs *t*-test, two-tailed, unequal variances). (C) *tub-AOX* rescue of developmental lethality from partial knockdown of Cox6b using *tub-GS* driver, as shown. Number of eclosing progeny at different concentrations of RU486, for the indicated numbers of *tub-AOX* transgenes. Means ± SD from ≥ 3 biological replicates. (D) Developmental delay of *tub-AOX*-rescued knockdown flies for Cox5a or

Cox5b, as shown. Means ± SD from ≥ 3 biological replicates, $p > 0.05$, comparing tub-AOX flies of each given sex, with and without concomitant knockdown of the indicated genes. (E) (Lack of) bang-sensitivity (square root of recovery time from mechanical shock) of 1 day-old flies rescued by *tub-AOX* from the lethality of Cox5b knockdown. All flies carried 4 *tub-AOX* transgenes, plus the *tub-GS* driver, and were cultured in the presence of 3 εM RU486, plus and minus the RNAi construct for Cox5b. These conditions generate larval-lethality in the absence of *tub-AOX*. Means ± SD for 12-15 individual flies of each sex and genotype analysed: the low numbers of eclosing progeny made it impractical to conduct this test on a larger scale. Maintaining Cox5b knockdown in the rescued flies after eclosion, by culturing them on food containing 200 εM RU486 for 19 d, also produced no bang-sensitivity (mean recovery time from vortexing 0.6 s, n = 35, was well within the range for wild-type flies). Survival over the first weeks of adult life was the same as in controls. (F) The rescued flies did, however, show a modest climbing defect, but since the number of rescued flies obtained using this procedure was both low and variable, the exact effect on locomotor activity could not be reliably quantified. The data presented thus represent a single such experiment. In this test, individual flies were assessed for their ability to climb 10 cm in 6 s, based on 3 trials. The bar graph shows the outcome for 12-15 individual flies of each sex and genotype
A2 analysed: the low numbers of eclosing progeny made it impractical to conduct this test on a larger scale.

Figure S3

Supplementary data on phenotypes and *UAS-AOX* rescue of cIV subunit knockdown

AOX rescue of lethality from (A) Cox4 or (B) Cox5b knockdown (*Drosophila* genes *CoIV*,
A1 *CoVb*), driven by *da-GAL4*. Proportion of eclosing progeny for different genotypes as shown, means ± SD from ≥ 3 biological replicates. Flies carrying the *Sb* balancer marker are those generated in the same cross, but which do not carry *da-GAL4* driver and thus have no knockdown. Note that, despite the very few adult flies eclosing, *UAS-AOX* enabled progeny to progress systematically to pupal stage (see Fig. 3A). *UAS-Ndi1* failed to bring about this

rescue, the progeny remaining as abnormal L1/L2 larvae as shown in the accompanying micrographs.

Figure S4

Supplementary data on tissue-restricted cIV knockdown and AOX rescue

A1

(A, C) Knockdown of (A) Cox6b or (C) Cox5b (*Drosophila* genes *CoVIb, CoVb*), using the drivers indicated. Flies with the CyO balancer marker are progeny from the same crosses, but without driver. Proportion of eclosing progeny of the genotypes indicated, as means $\pm$ SD for $\geq$ 3 biological replicates. (B) Absence of bang-sensitivity of flies knocked down for Cox5b using the drivers indicated. Means $\pm$ SD of square root of recovery time from mechanical shock, for batches of 10 individual flies of each sex and genotypes shown. ((D, E) Numbers of flies eclosing, of the indicated genotypes. Note that, in the experiment shown in panel E, progeny males carry a wild-type X chromosome, whilst females carry a copy of the *elav*-GAL4 (458) driver). Means $\pm$ SD of $\geq$ 3 biological replicates. Asterisks indicate significant differences (*t*-test, $p < 0.05$) from corresponding male flies without the driver and from females also expressing AOX. (E) Micrographs showing typical phenotypes of flies of the indicated genotypes (X, 2, 3 indicate wild-type chromosomes. Without AOX, flies knocked down for Cox4 by the *elav*-GAL4 (458) driver fail to eclose, whilst those ŕrescuedô by AOX do eclose successfully and some inflate their wings, but nevertheless drown in the food unless transferred immediately to empty vials.

Figure S5

Expression patterns conferred by different GAL4 drivers

Each driver was combined with UAS-GFP (Stinger or, where indicated, mCD8), and progeny analyzed at different developmental stages by fluorescence microscopy, with dissection as indicated. Comparable magnifications are shown, though some images have been resized and/or cropped for clarity. In some images signal has been optimized for brightness and contrast, but no gamma correction or any other manipulations were carried out. (A) Driver

BG57 gives expression in specific muscle and salivary gland nuclei at L2 larval stage (note background fluorescence from gut) plus mainly abdominal signal during pupal stage, with clear expression in specific abdominal muscles that persists into adult stage: Note that the adult images show the same individual fly, but with addition of visible light (bottom left), to clarify the region of expression. (B) Driver G14 gives expression in all muscle nuclei in L1 and L2 larvae. In adults expression is seen in the major muscles of the thorax and the most anterior part of the abdomen. (C) Different neuronal drivers give similar expression patterns in L1 larvae (nuclei of brain, ventral nerve cord and peripheral neurons), but of different intensities. Expression from *nrv2*-GAL4 is weak, being comparable with the background fluorescence from the gut (left hand images), whilst the *elav*-GAL4 drivers (right-hand panels, as indicated) give stronger expression. (D) *nrv2*-GAL4-driven expression remains weak at L2 and in pupal stage, again comparable with background fluorescence from gut or cuticle. (E) *elav*-GAL4 driver strains give prominent expression in CNS and salivary gland nuclei at larval stage L3. (F) In stripped pupae, *elav*-GAL4 driver 458 gives clear expression in the region of the major thoracic muscles. Fluorescence is clearly seen in dissected thoracic muscle and in the CNS, as indicated. (G) Expression in the CNS and weakly in thoracic muscle, driven by *elav*-GAL4 (458) is maintained in dissected adult organs, as well as in testis and in specific regions of the gut. (H) In addition to signal in the CNS, very weak fluorescence is seen in a subset of thoracic muscles, driven by *elav*-GAL4 strain 8760. Visible light images show the dissected muscle in which fluorescence was very weak (bottom right panels) or undetectable (top right panels). (I) In addition to signal in the CNS, weak fluorescence is seen in the anterior abdomen in adults, driven by *elav*-GAL4 strain 8760. Only very weak fluorescence is seen in dissected thoracic muscle (right), shown alongside the visible light image.

Figure S6

Analysis of *elav*-GAL4-driven expression by immunocytochemistry and histochemistry

Comparable magnifications are shown, though some images have been resized and/or cropped for clarity. In some images signal has also been adjusted for brightness and contrast, but no gamma correction or any other manipulations were carried out. (A-D) Immuocytochemistry using the indicated antibodies, with counterstaining by DAPI (A, B), in sections from the tissues of adult flies of the indicated genotypes. (A) *elav*-GAL4 (458) drives expression of AOX in a specific subset of cells in the brain, as well as (B) thoracic muscle (image shows prominent staining in extracoxal muscle). Note absence of any detectable signal in wild-type (wt) flies. Note that in merged (DAPI + antibody) images overexposure of DAPI fluorescence masks some of the antibody fluorescence, (C, D) *elav-GAL4* (458) also drives knockdown of

A1

cIV (Cox4, *CoIV* gene or Cox5b, *CoVb*, gene) in a subset of cells in the brain (C), as well as in thoracic muscle (D), based on comparison of signal from antibodies against ATP synthase Ŭ(marker for cV) and COX4 (marker for cIV). (E, F) Histochemical staining of thoracic muscle sections from flies of the indicated genotypes, for COX/SDH, COX alone or SDH alone, as indicated. Note that, in our hands, SDH signal was very weak when implementing the standard procedure for dual staining; SDH staining was therefore carried out on parallel sections.

Figure S7

A2

B4

Residual phenotypes following Ndi1 rescue of cI knockdown

(A) Developmental time and (B) bang-sensitivity of flies of the indicated genotypes, ages and sex, reared at 25 °C. Means ± SD of 3 biological replicates; bang-sensitivity data generated by analysis of batches of >50 individual flies of each class. Note that *UAS-Ndi1* transgene is present in all flies shown in panel (A). Where indicated (*), $p < 0.01$ (Student's *t* test, 2-tailed, unequal variances), comparing flies of the given sex knocked down for the indicated gene, compared with each group of corresponding control flies (driver only, RNAi transgene only, or neither). These experiments make use of the previous observation that co-expression of

Ndi1 was able to complement the larval lethality of global knockdown of either of two single-copy genes for cI subunits, namely Ndufa8 (mouse nomenclature: *Drosophila* gene *CG3683*) or Ndufa9 (*Drosophila* gene *CG6020*), driven by *da-GAL4* (20). The rescued flies, in both cases, eclosed with a long developmental delay (3-5 d, panel A) and were mildly (but significantly) bang-sensitive when first tested at room temperature the day after eclosion (panel B). When mated with wild-type flies of the opposite sex, the rescued females were fertile, giving 24.5 $\pm$ 5.1 and 17.8 $\pm$ 6.1 progeny from individual females rescued from knockdown of CG3683 and CG6020, respectively. The resulting larvae appeared weak, and during wandering stage did not emerge more than 1-3 cm from the food before pupariation. However, all pupae eclosed and the resulting adults appeared normal. In contrast, rescued males produced no progeny (>50 Ndi1-rescued males tested from knockdown of each gene). Testis morphology of the rescued males appeared grossly normal (phase-contrast microscopy, panel C), although knockdown was clearly effective in testis, as judged by the levels of the cI marker protein NDUFS3 in Western blots (panel D). When testes from the Ndi1-rescued males were punctured by dissection needles, superficially mature sperm were seen, but were much less plentiful than from control males, and were immotile when exposed to standard buffer, whereas sperm from control males processed in parallel were motile. In (D) all flies carry the $Ndi1^{A46}$ transgene, but there is expression only where knockdown of the indicated genes is co-driven by *da-GAL4*. Molecular weights extrapolated from markers; the separate panels are from nonadjacent tracks of the same gel.

Figure S8

Supplementary data on modification of *levy*^{1} and Cox6a-knockdown phenotypes

(A-C) Lifespan curves of *levy*^{1} flies also carrying various transgenes as indicated. Plotted data represent batches of $\geq$ 100 flies of each sex and genotype indicated. Mean lifespans as indicated. Neither *UAS-AOX*/*da-GAL4*, nor 4 copies of *tub-AOX*, lengthened the short lifespan of *levy*^{1} flies. (D) *UAS-Ndi1* control for *UAS-AOX* rescue of seizure-sensitivity of 7-day old *levy*^{1} flies at 29 ºC. Means $\pm$ SD of square root of recovery time from mechanical

shock, for batches of ≥ 50 individual flies of the genotypes indicated. Use of the *UAS-Ndi1* transgene in place of *UAS-AOX* (see Fig. 6B) failed to bring about rescue, instead exacerbating the phenotype, p <0.001 (Studentôs *t*-test, two-tailed, unequal variances). (E) Uninflated wings phenotype produced by Cox6a knockdown (*levy* gene) using *elav-GAL4* driver strain 458, with rescue by *UAS-AOX*.

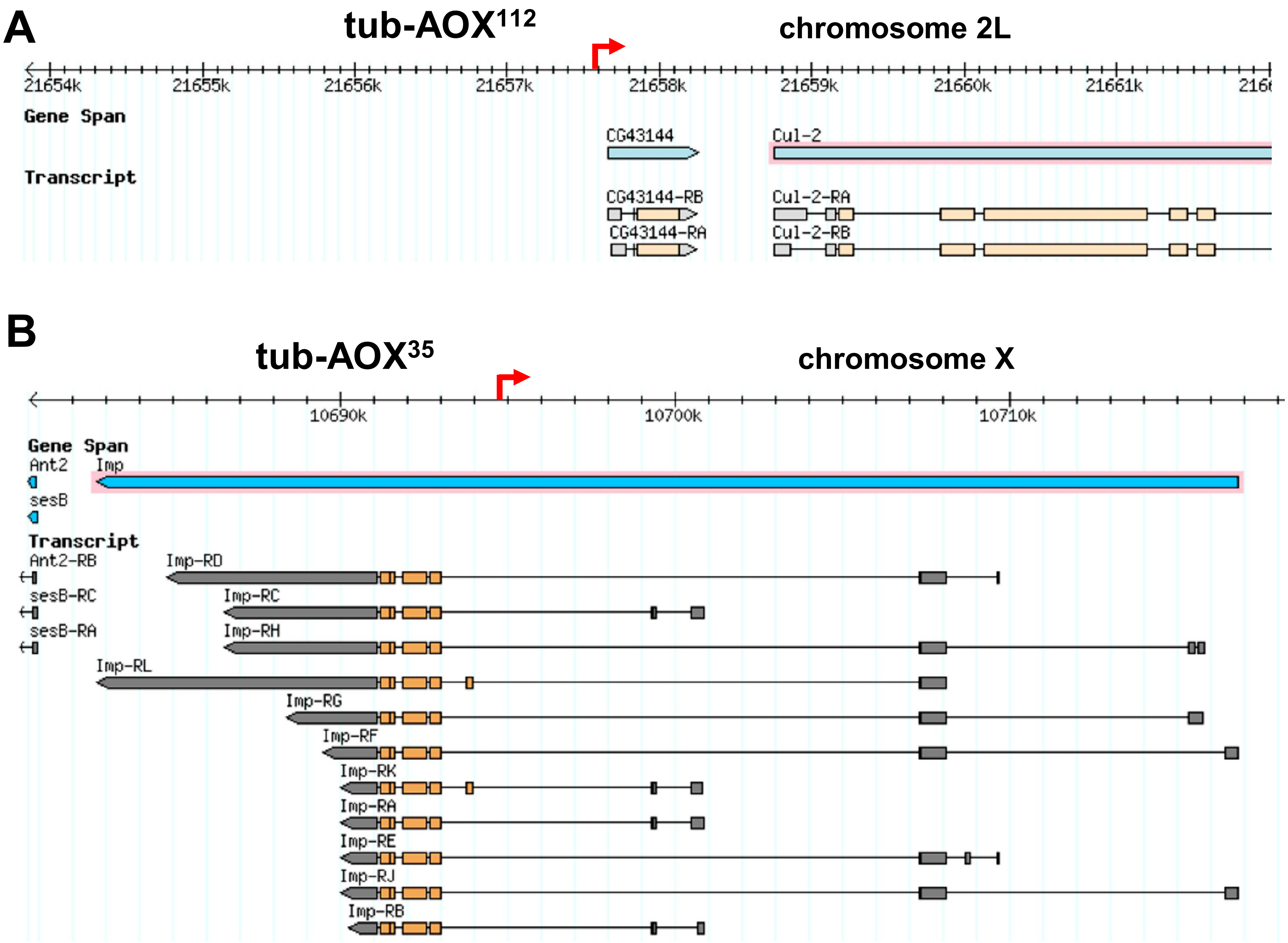

Figure S1, Kemppainen *et al*, page 1 of 3

C

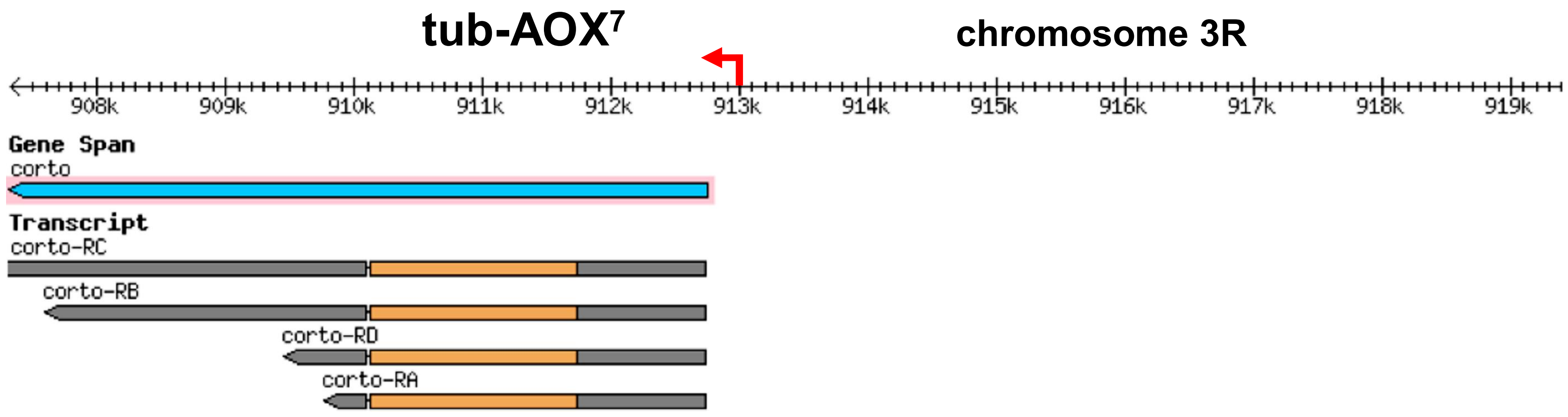
tub-AOX7
chromosome 3R
908k
909k
910k
911k
912k
913k
914k
915k
916k
917k
918k
919k
Gene Span
corto
Transcript
corto-RC
corto-RB
corto-RD
corto-RA

D

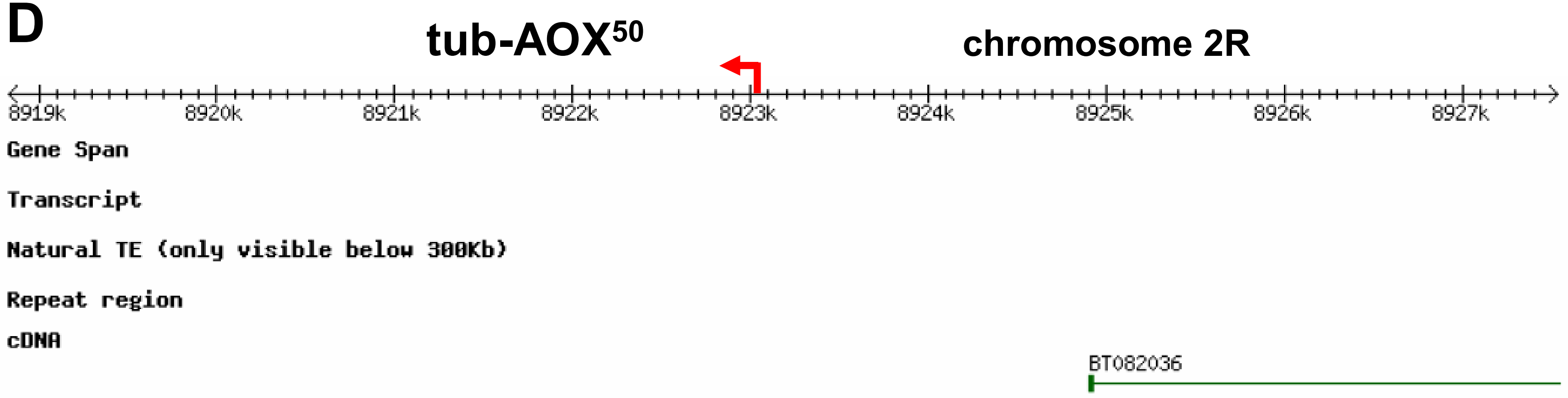
tub-AOX50
chromosome 2R
8919k
8920k
8921k
8922k
8923k
8924k
8925k
8926k
8927k
Gene Span
Transcript
Natural TE (only visible below 300Kb)
Repeat region
cDNA
BT082036

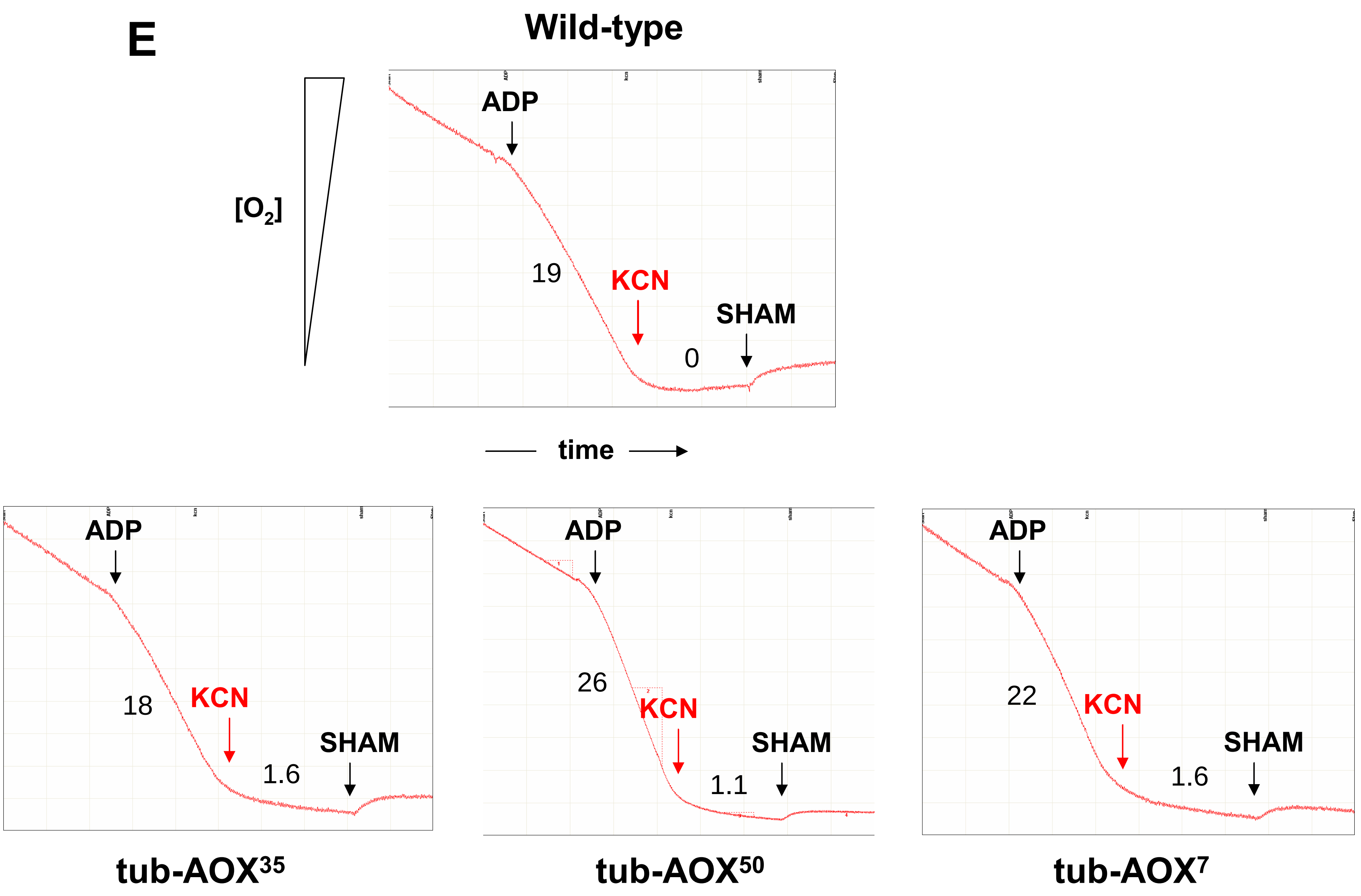
E
Wild-type
[O2]
ADP
19
KCN
SHAM
0
time
ADP
18
KCN
1.6
SHAM
tub-AOX35
ADP
26
KCN
1.1
SHAM
tub-AOX50
ADP
22
KCN
1.6
SHAM
tub-AOX7

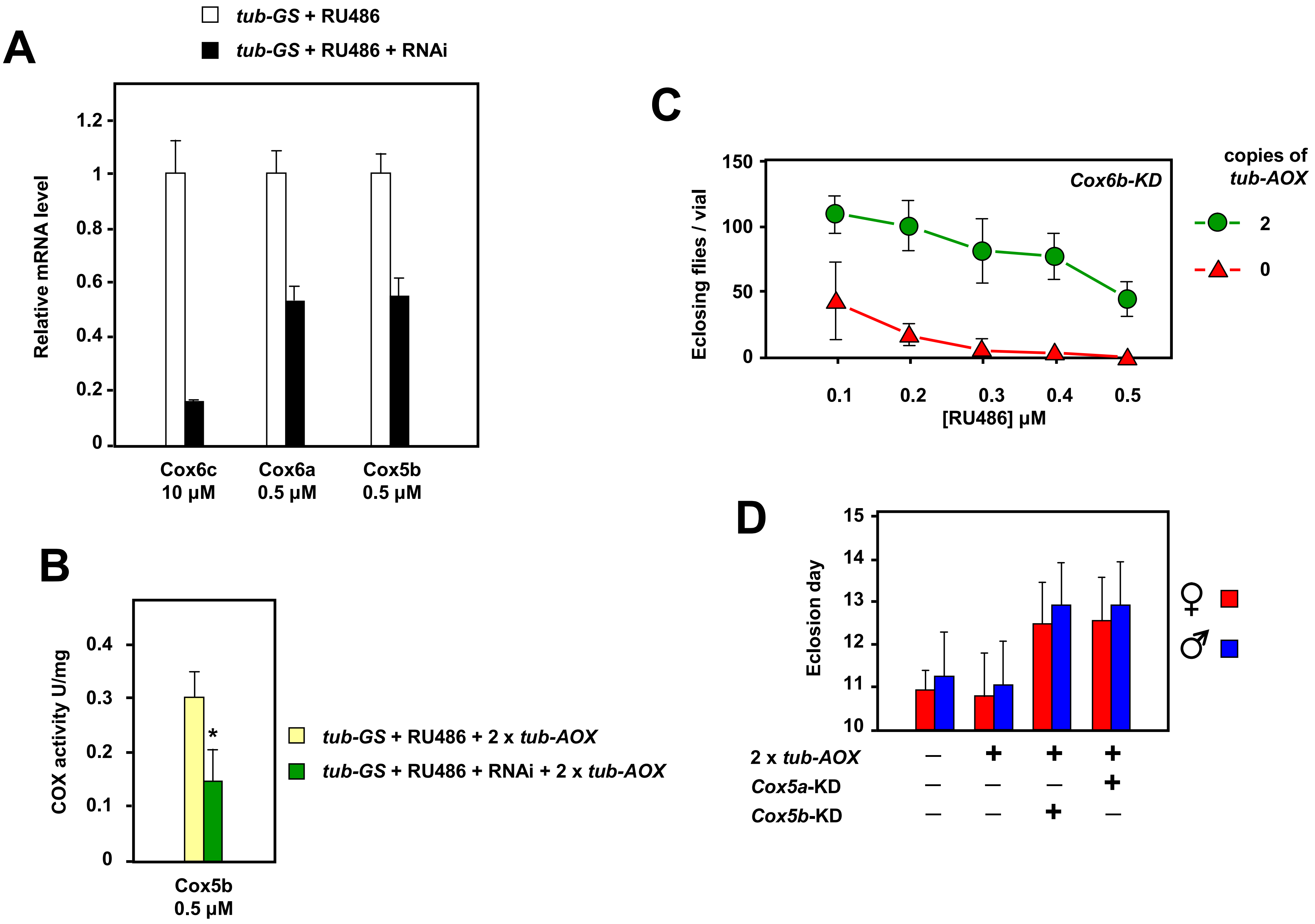

Figure S2, Kemppainen *et al*, page 1 of 2

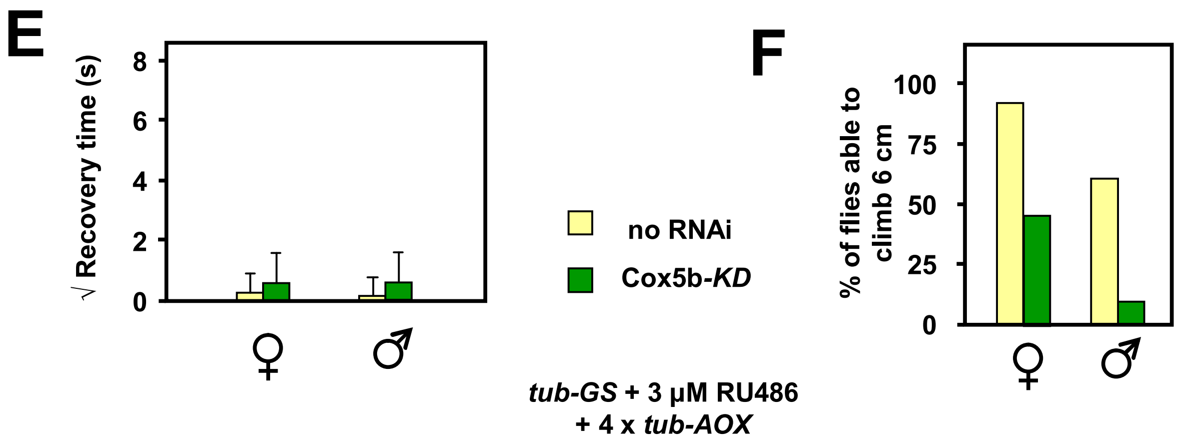

Figure S2, Kemppainen *et al*, page 2 of 2

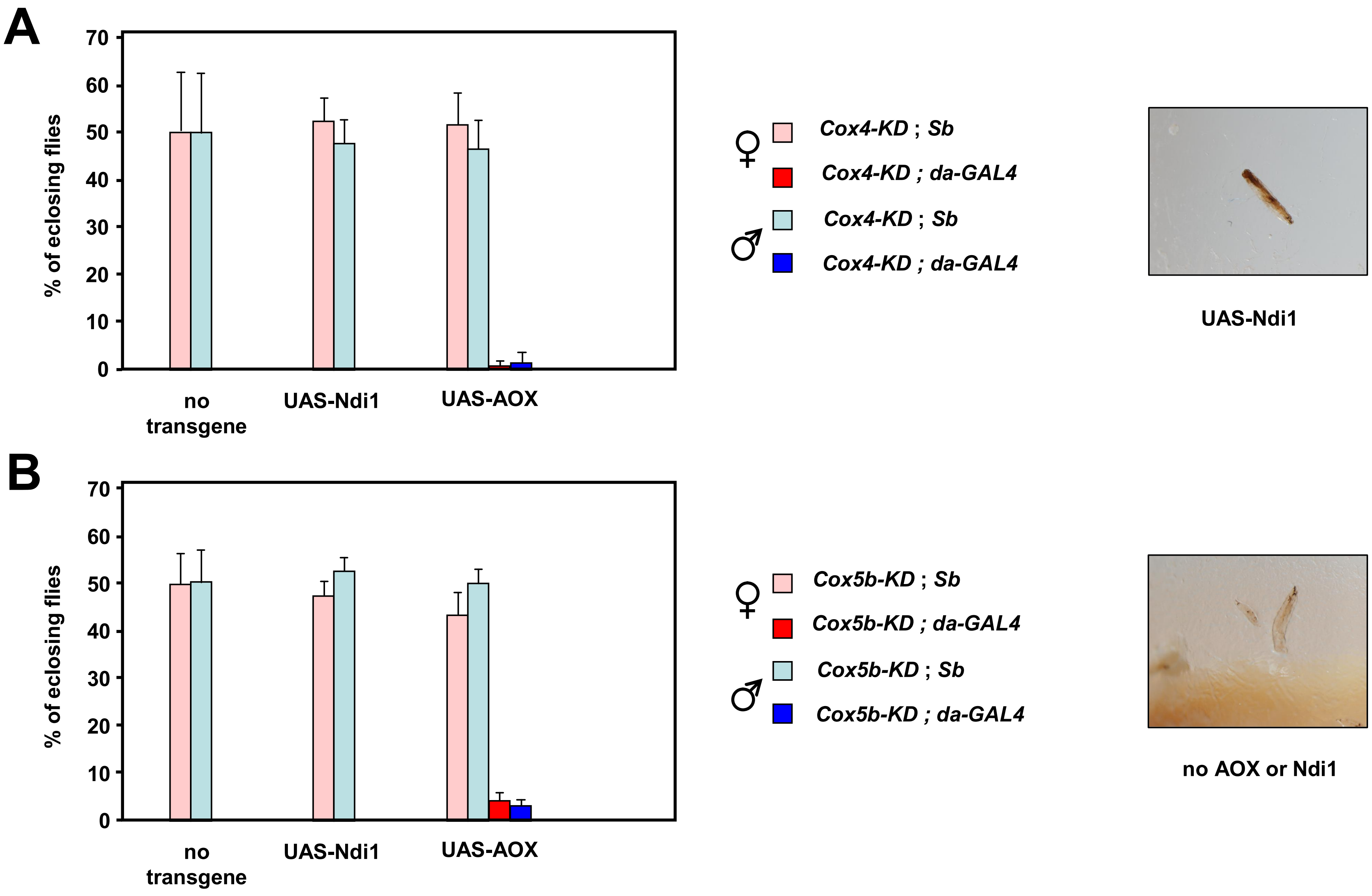

Figure S3, Kemppainen *et al*

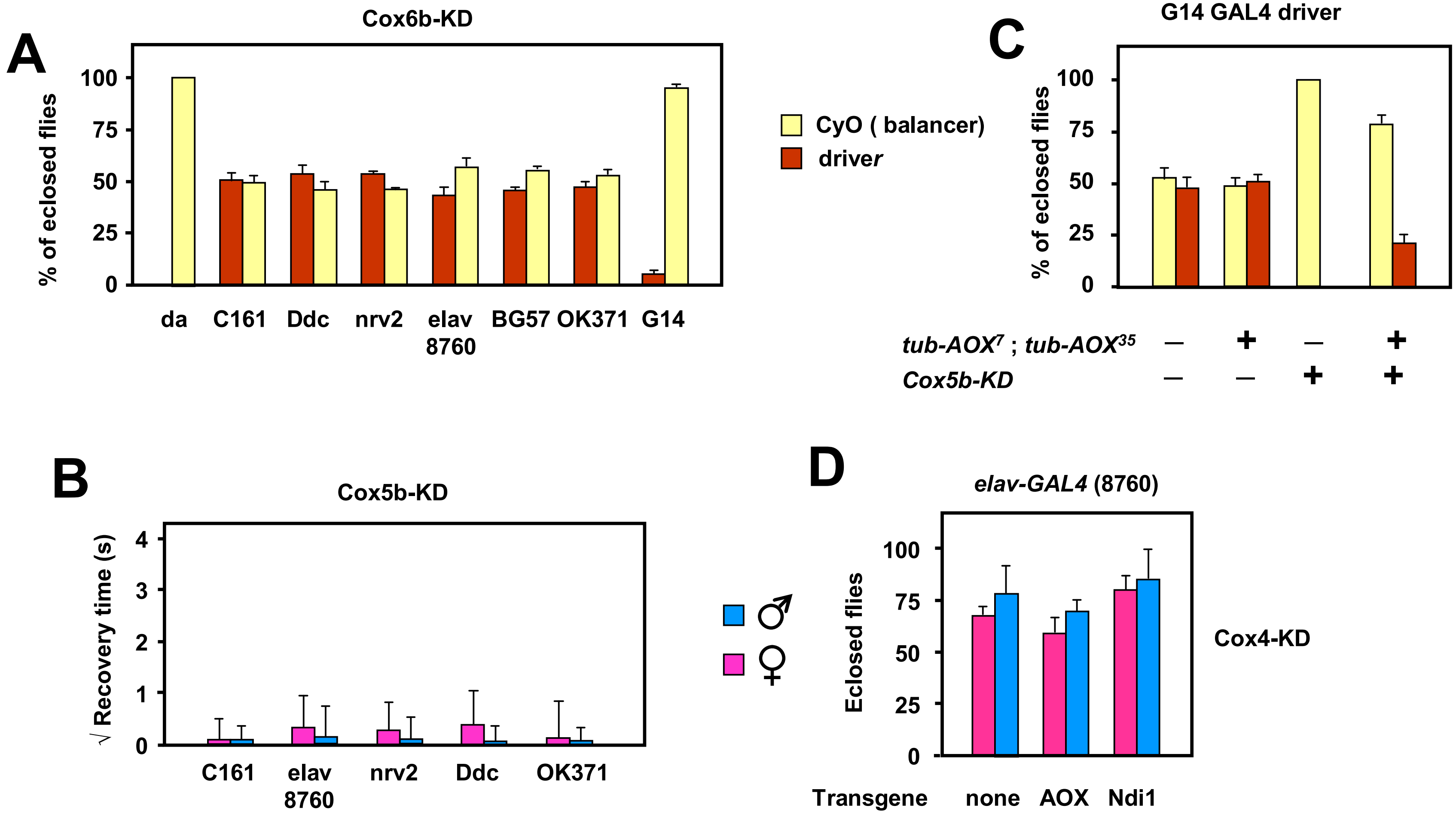
A
Cox6b-KD
% of eclosed flies
100
75
50
25
0
da
C161
Ddc
nrv2
elav
8760
BG57
OK371
G14
CyO (balancer)
driver
C
G14 GAL4 driver
% of eclosed flies
100
75
50
25
0
tub-AOX7 ; tub-AOX35
Cox5b-KD
B
Cox5b-KD
√ Recovery time (s)
4
3
2
1
0
C161
elav
8760
nrv2
Ddc
OK371
♂
♀
D
elav-GAL4 (8760)
Eclosed flies
100
75
50
25
0
Transgene
none
AOX
Ndi1
Cox4-KD

E

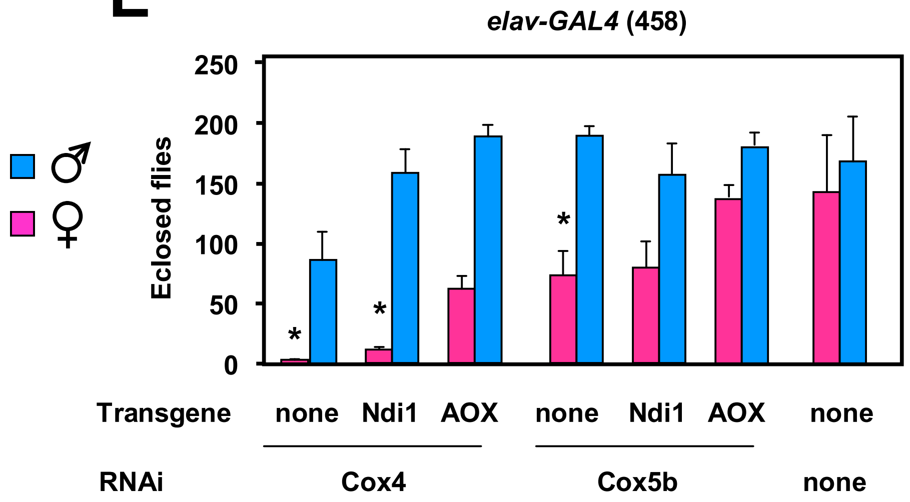

F

elav-GAL4 (458) : *Cox4*-KD / 2

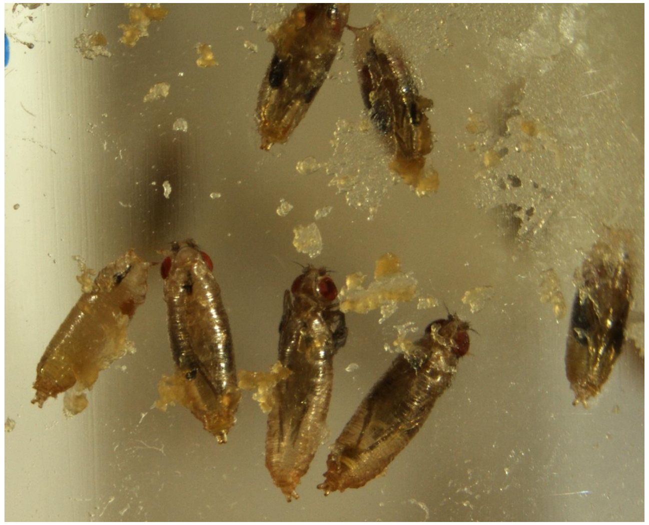

failure of eclosion

elav-GAL4 (458) : *Cox4*-KD / 2 ; *UAS-AOX*^{F6} / 3

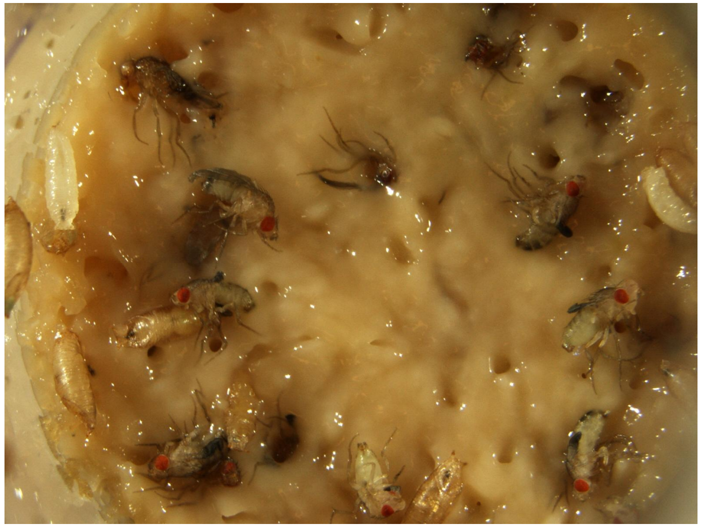

weak eclosed adults dying in food

Figure S4, Kemppainen *et al*, page 2 of 2

A

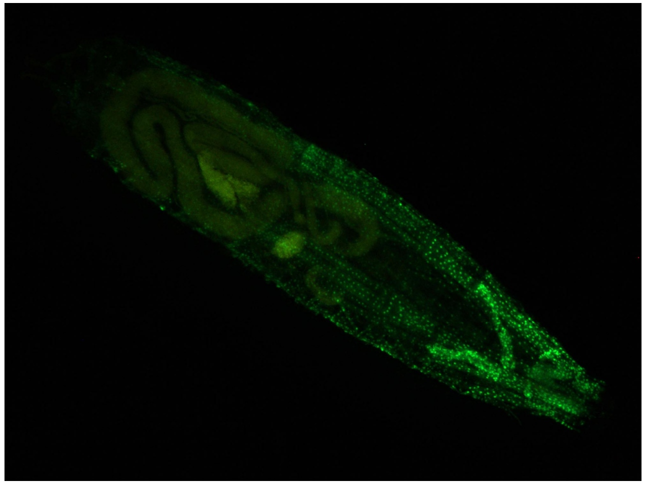

L2 larva

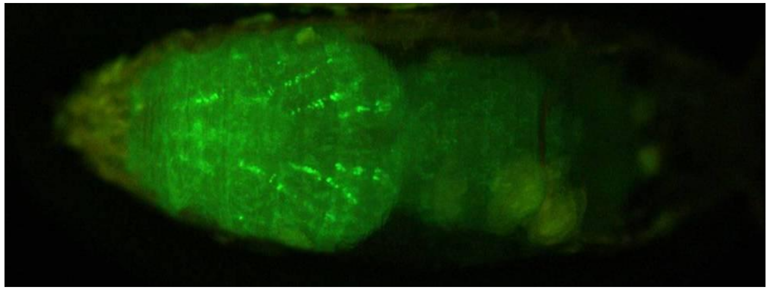

late pupa

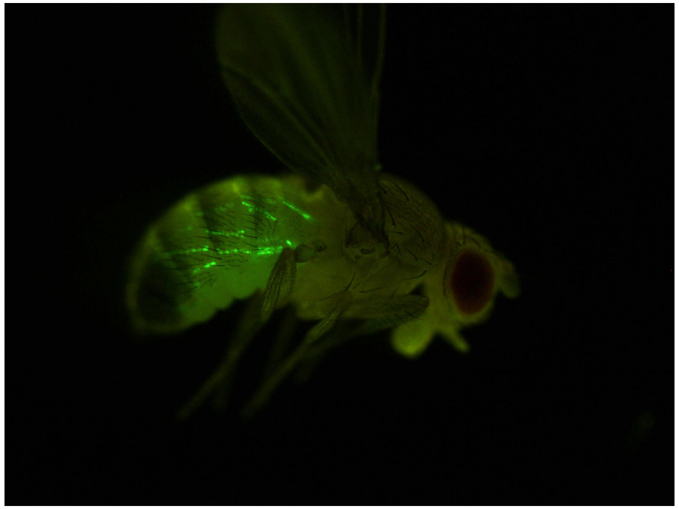

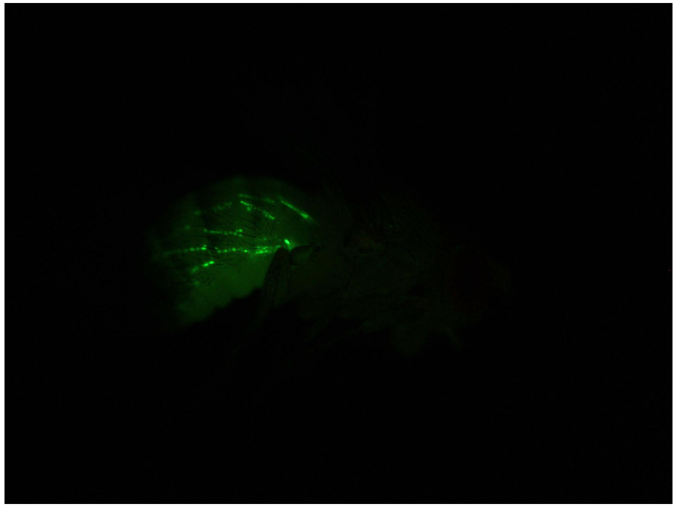

Adult male

BG57 GAL4 driver

B

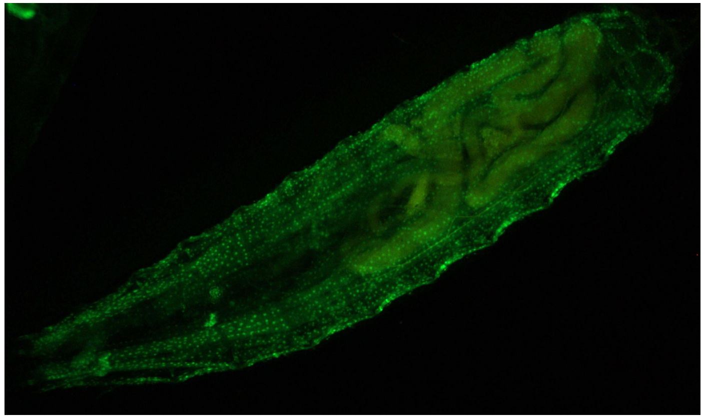

L1 larva

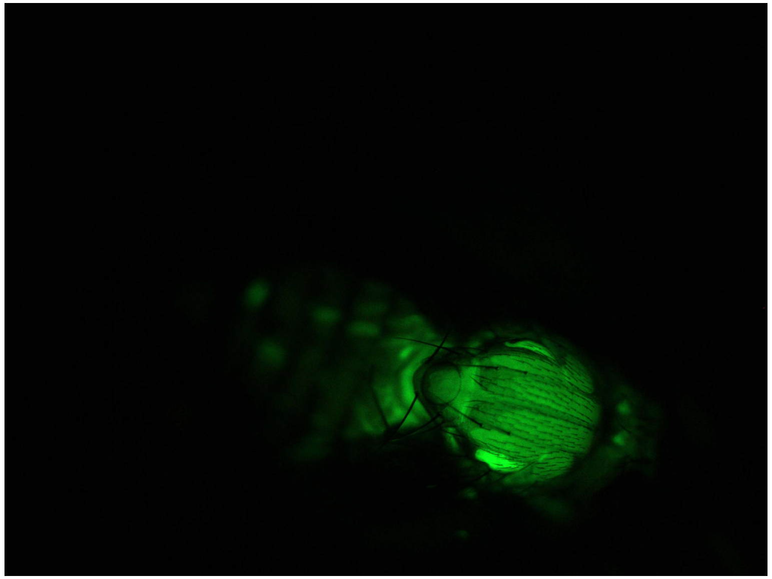

Adult female

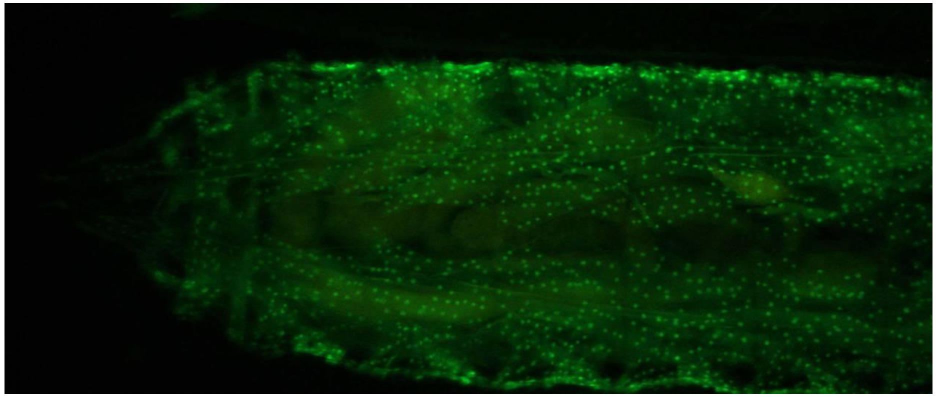

L2 larva

G14 GAL4 driver

C

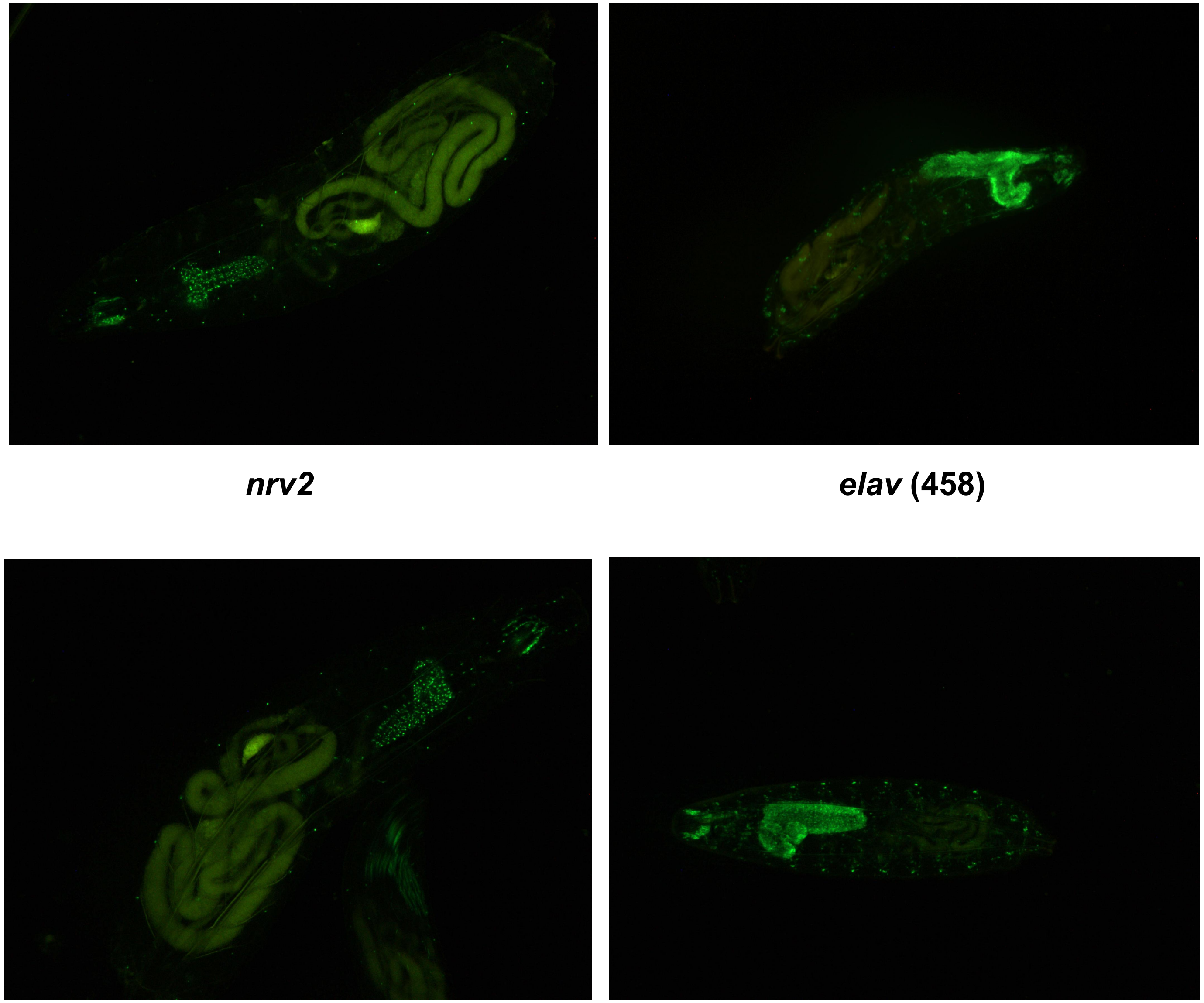

nrv2 **|** ***elav*** **(458)**

L1 larvae

nrv2 **|** ***elav*** **(8760)**

D

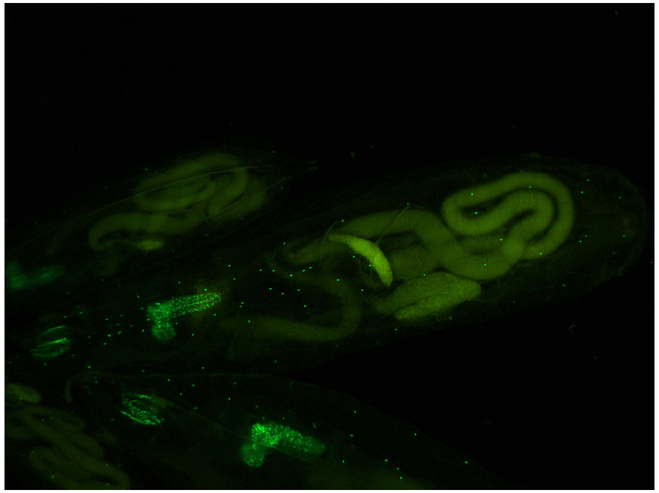

L2 larvae

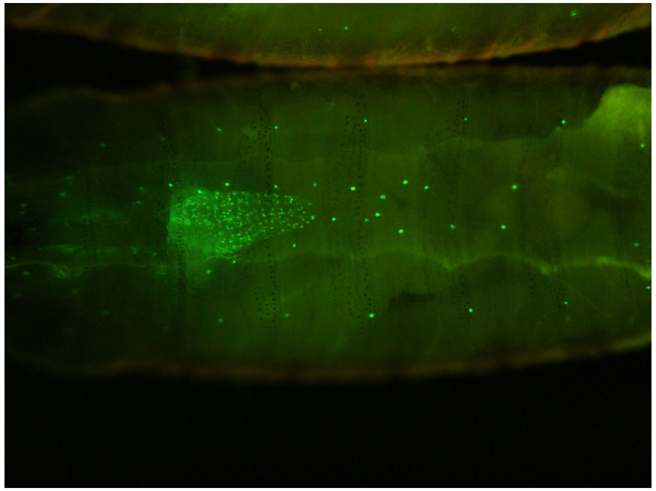

early pupa

nrv2-GAL4

E

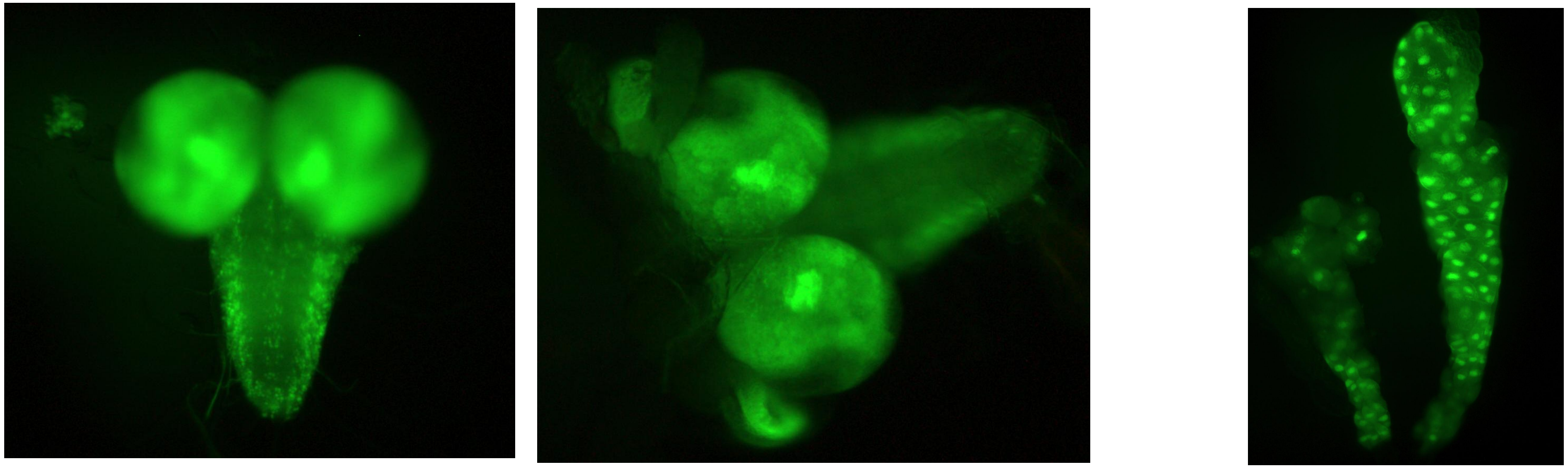

dissected CNS

elav (458) L3 larvae

dissected
salivary gland

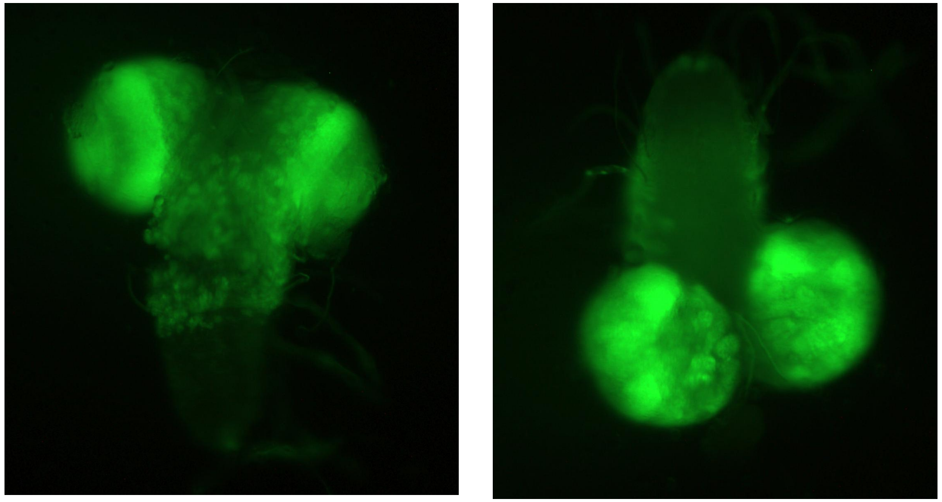

elav (8760) L3 larvae

dissected CNS

F

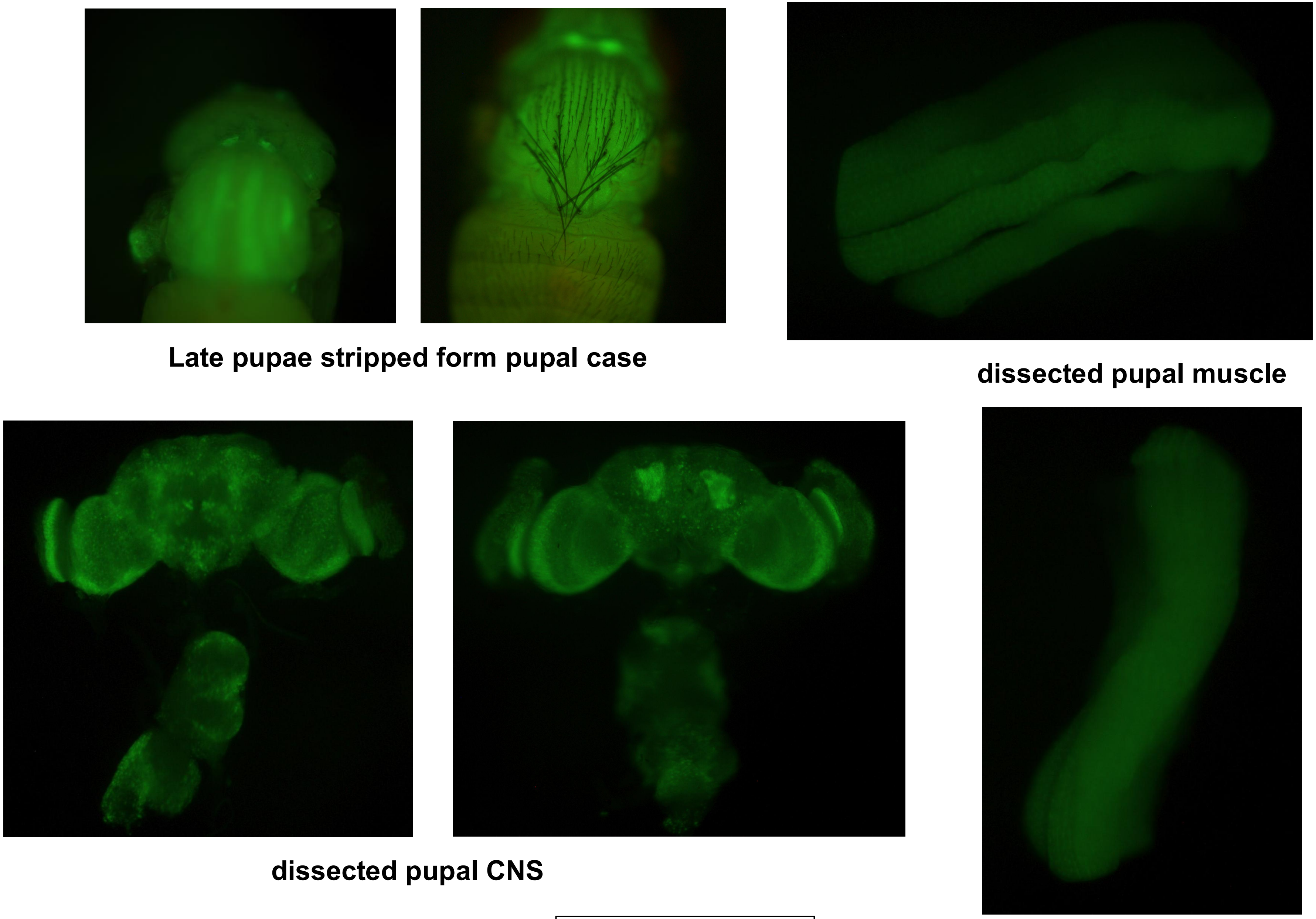

elav (458) pupae

Figure S5, Kemppainen *et al*, page 6 of 9

G

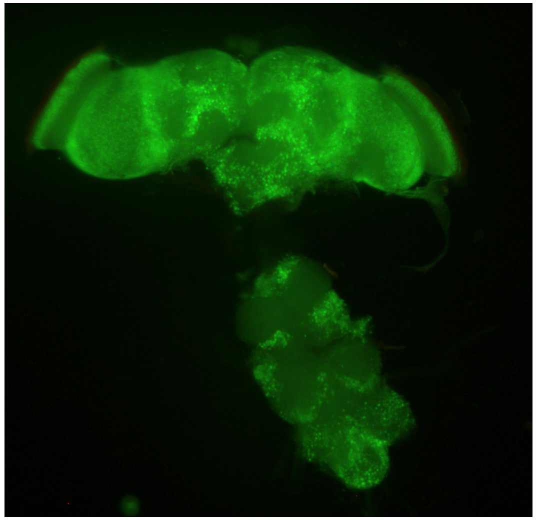

dissected adult CNS

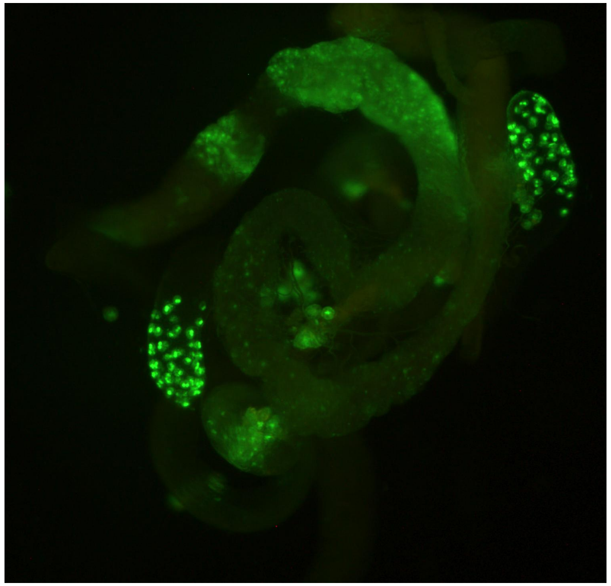

dissected adult gut and testis

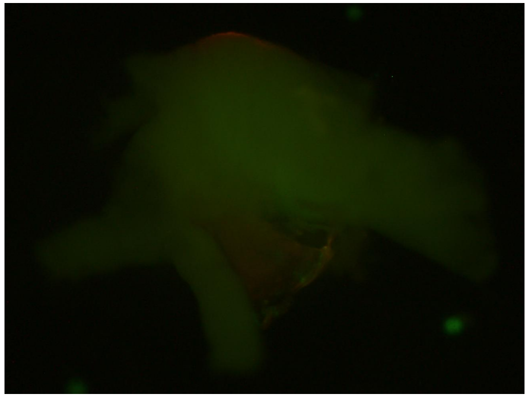

dissected adult thoracic muscle

***elav* (458) adults**

H

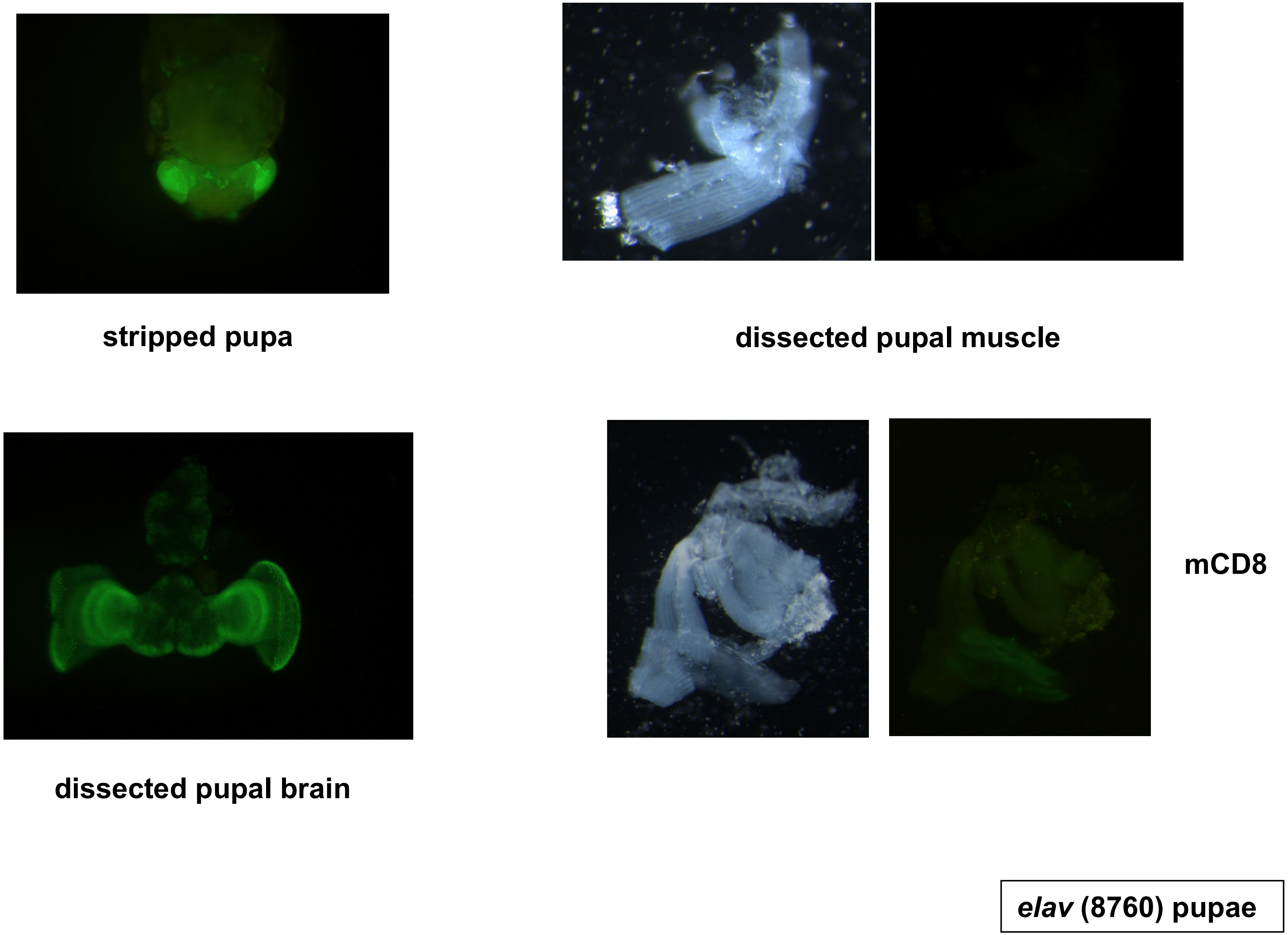
stripped pupa
dissected pupal muscle
dissected pupal brain
mCD8
elav (8760) pupae

I

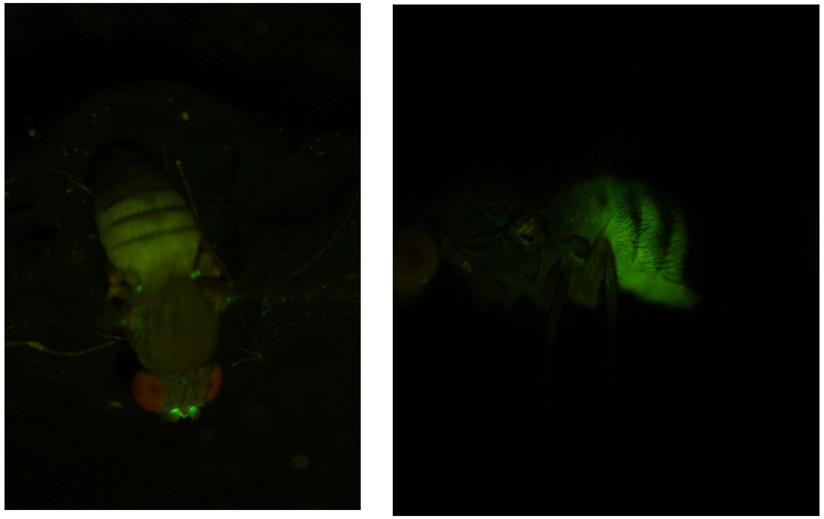

adult male

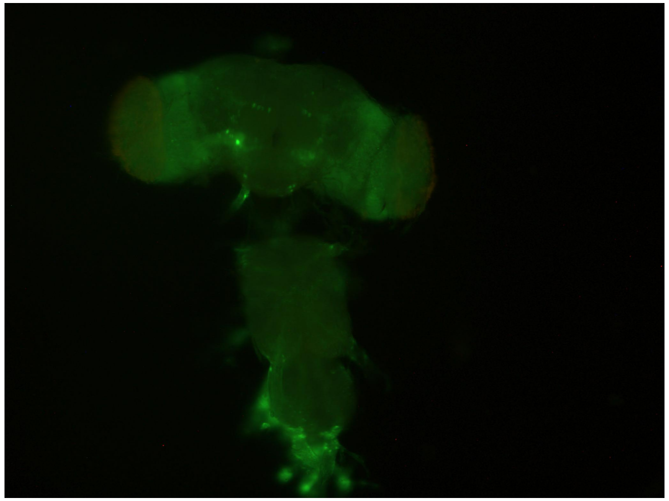

dissected adult brain

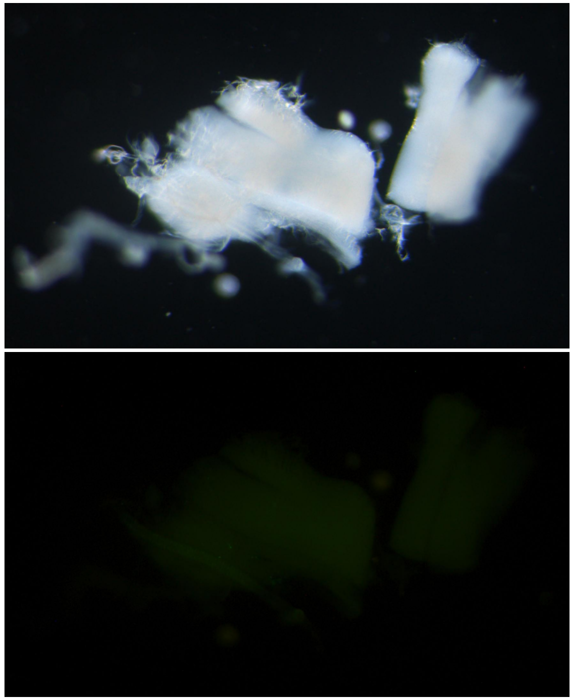

dissected adult muscle

***elav* (8760) adults**

A

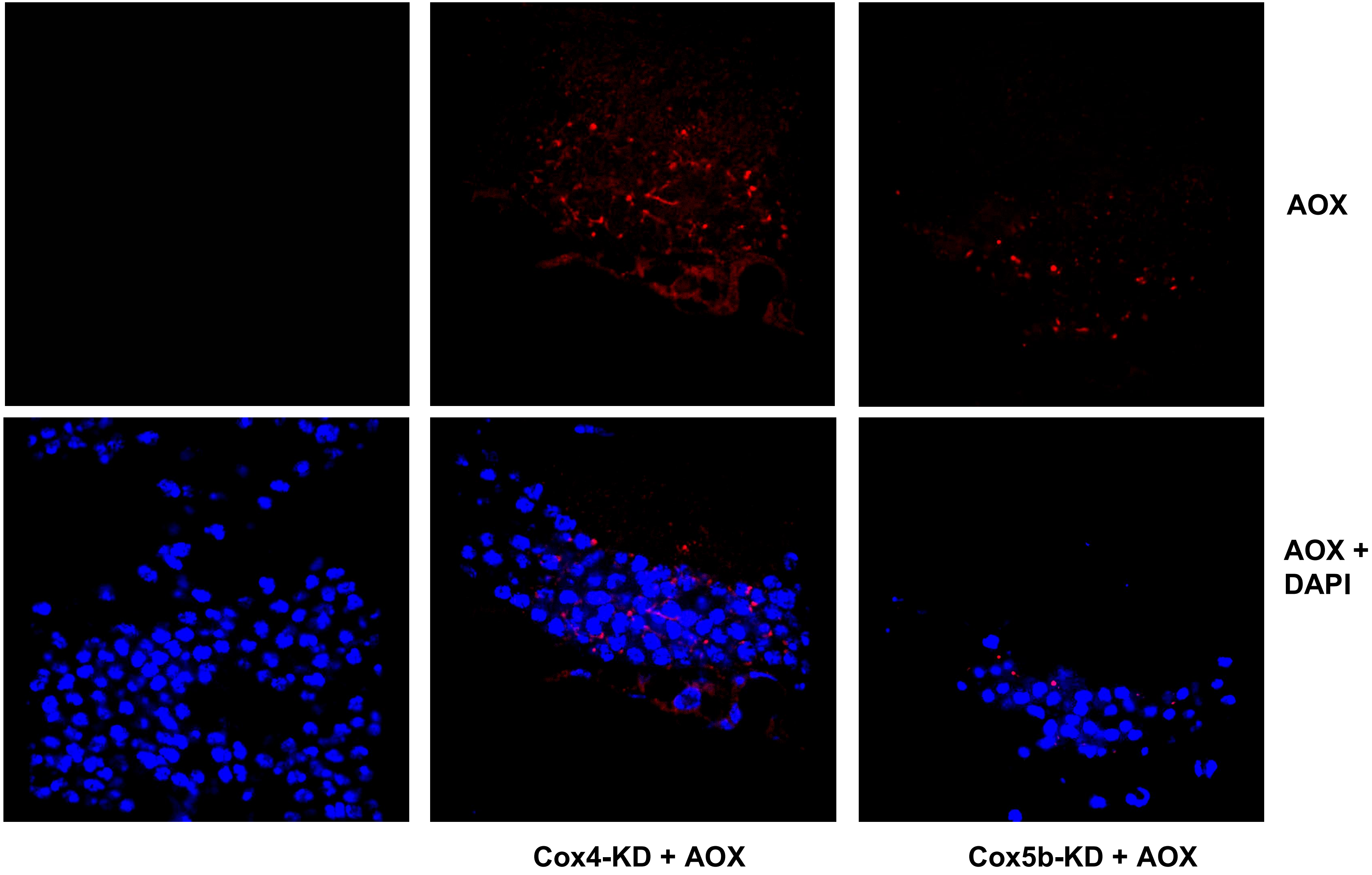

elav-GAL4 (458) – brain

B

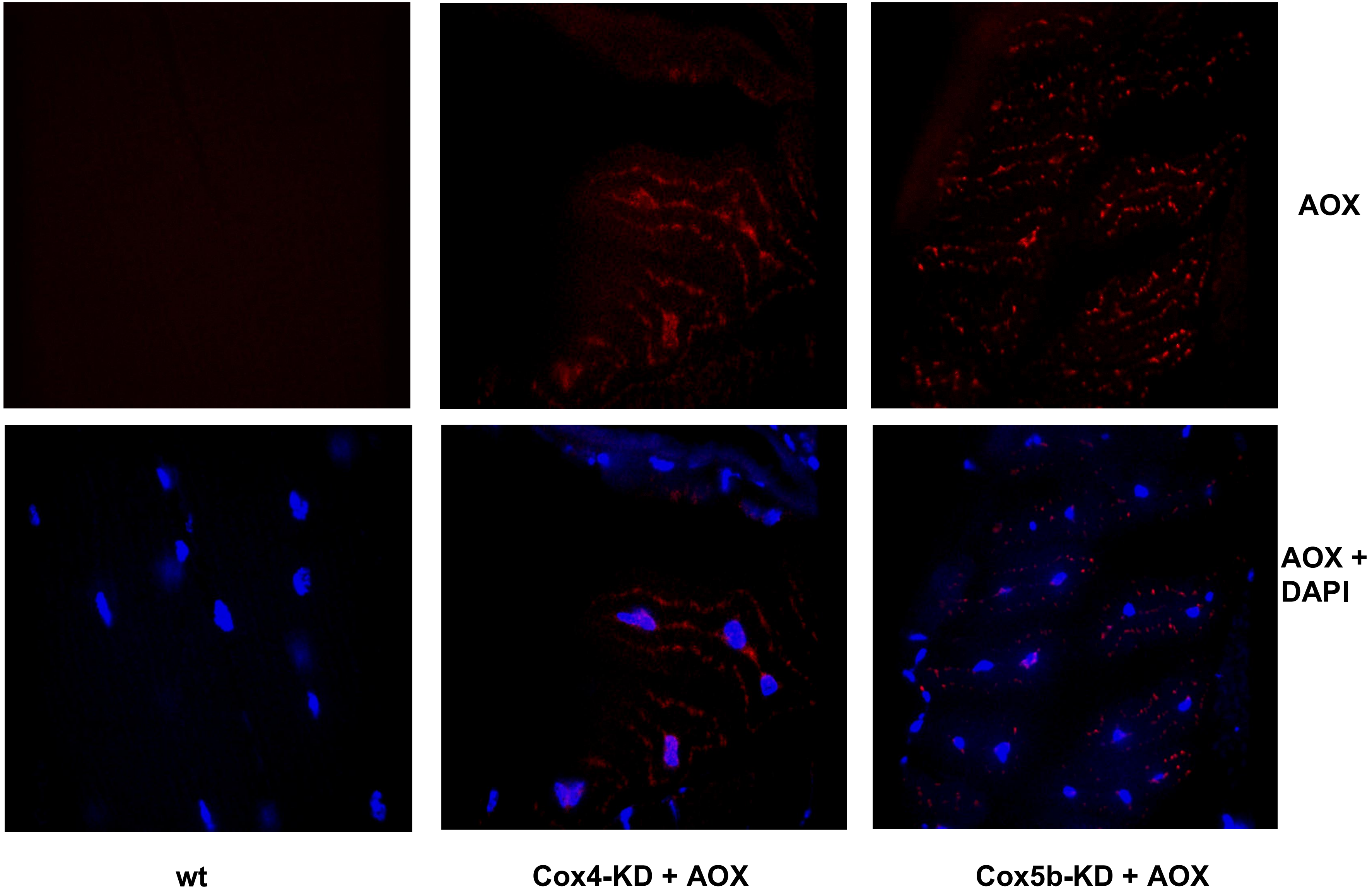

elav-GAL4 (458) – muscle

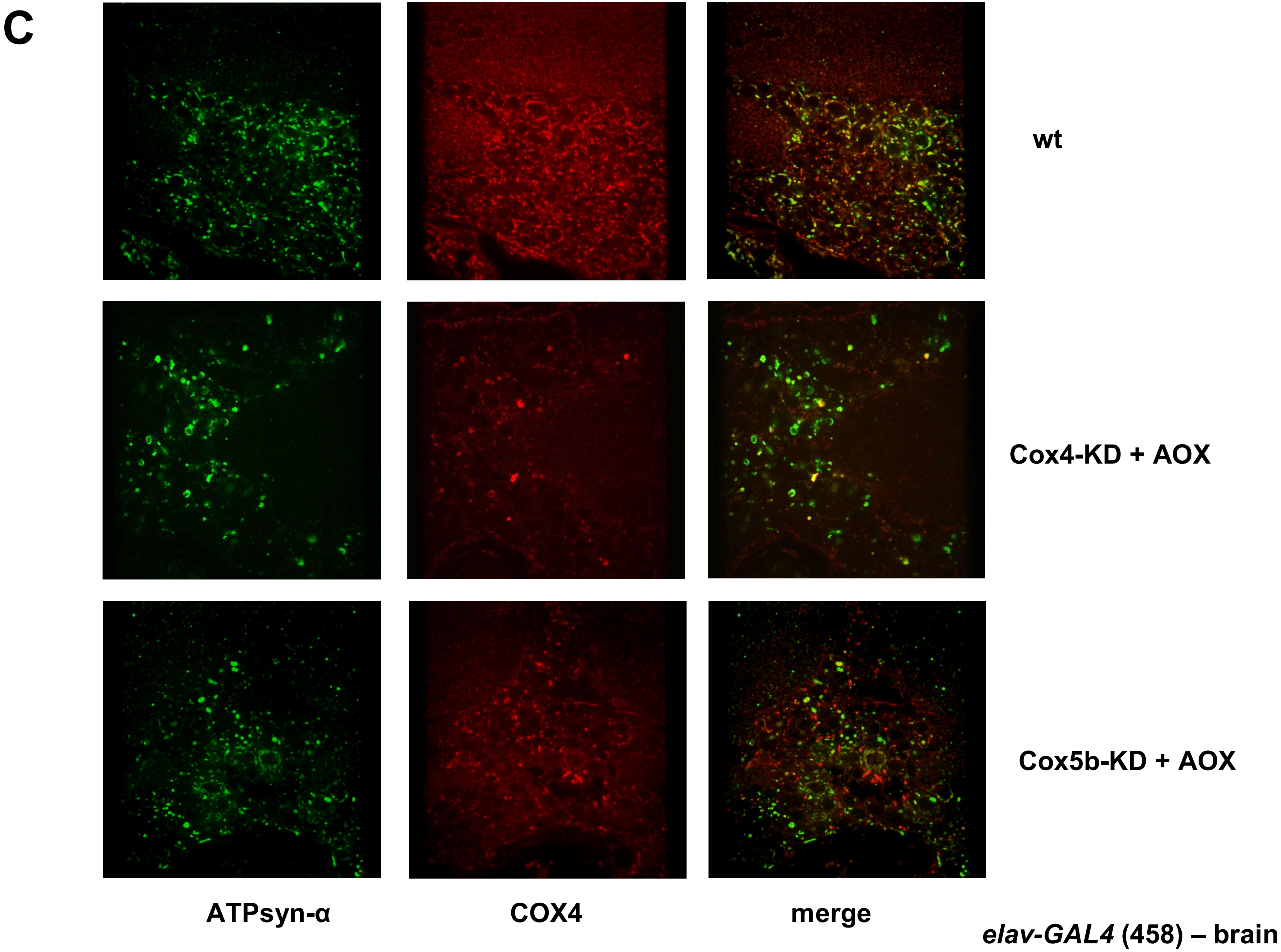
C
wt
Cox4-KD + AOX
Cox5b-KD + AOX
ATPsyn-α
COX4
merge
elav-GAL4 (458) – brain

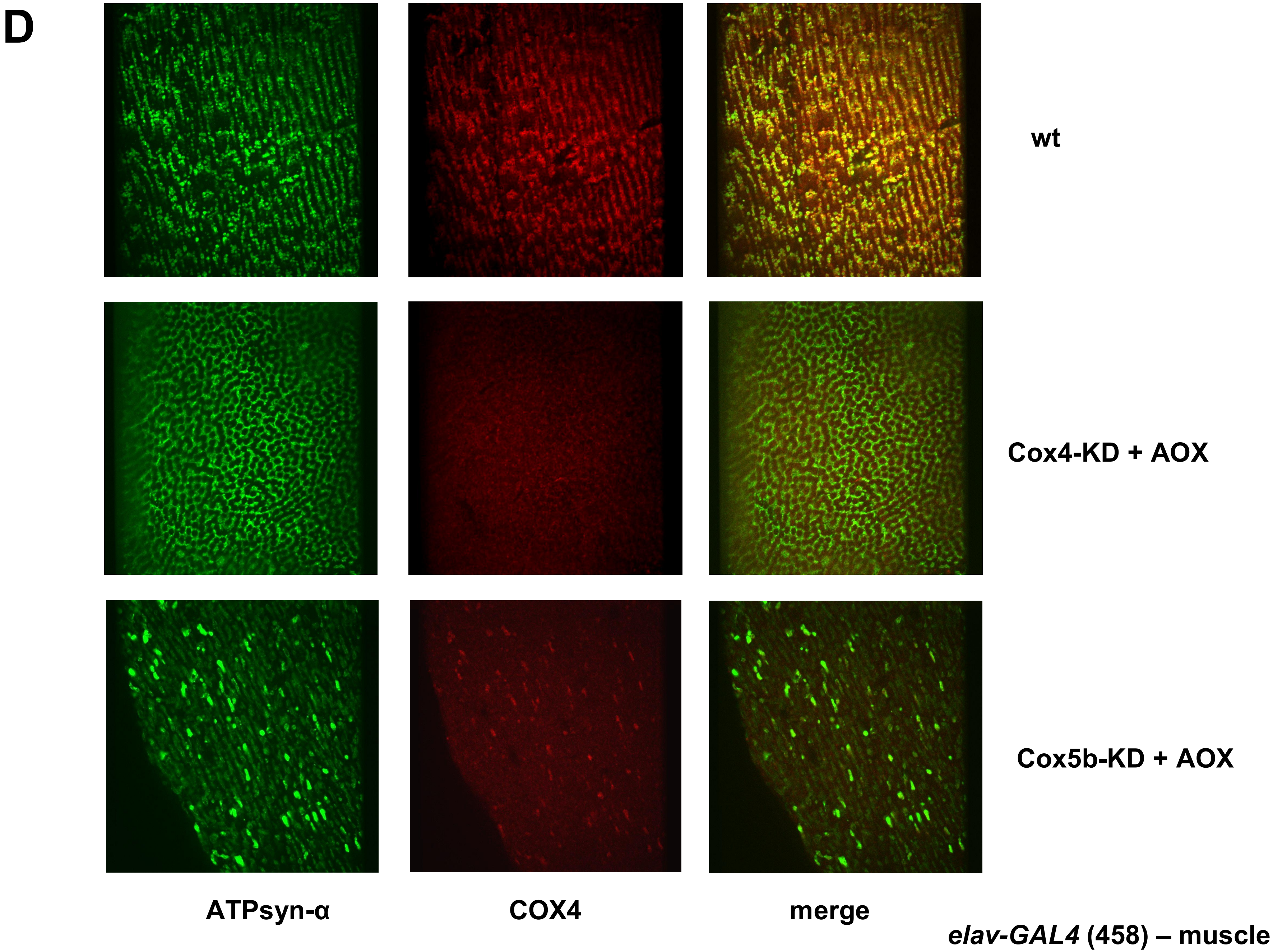
D
wt
Cox4-KD + AOX
Cox5b-KD + AOX
ATPsyn-α
COX4
merge
elav-GAL4 (458) – muscle

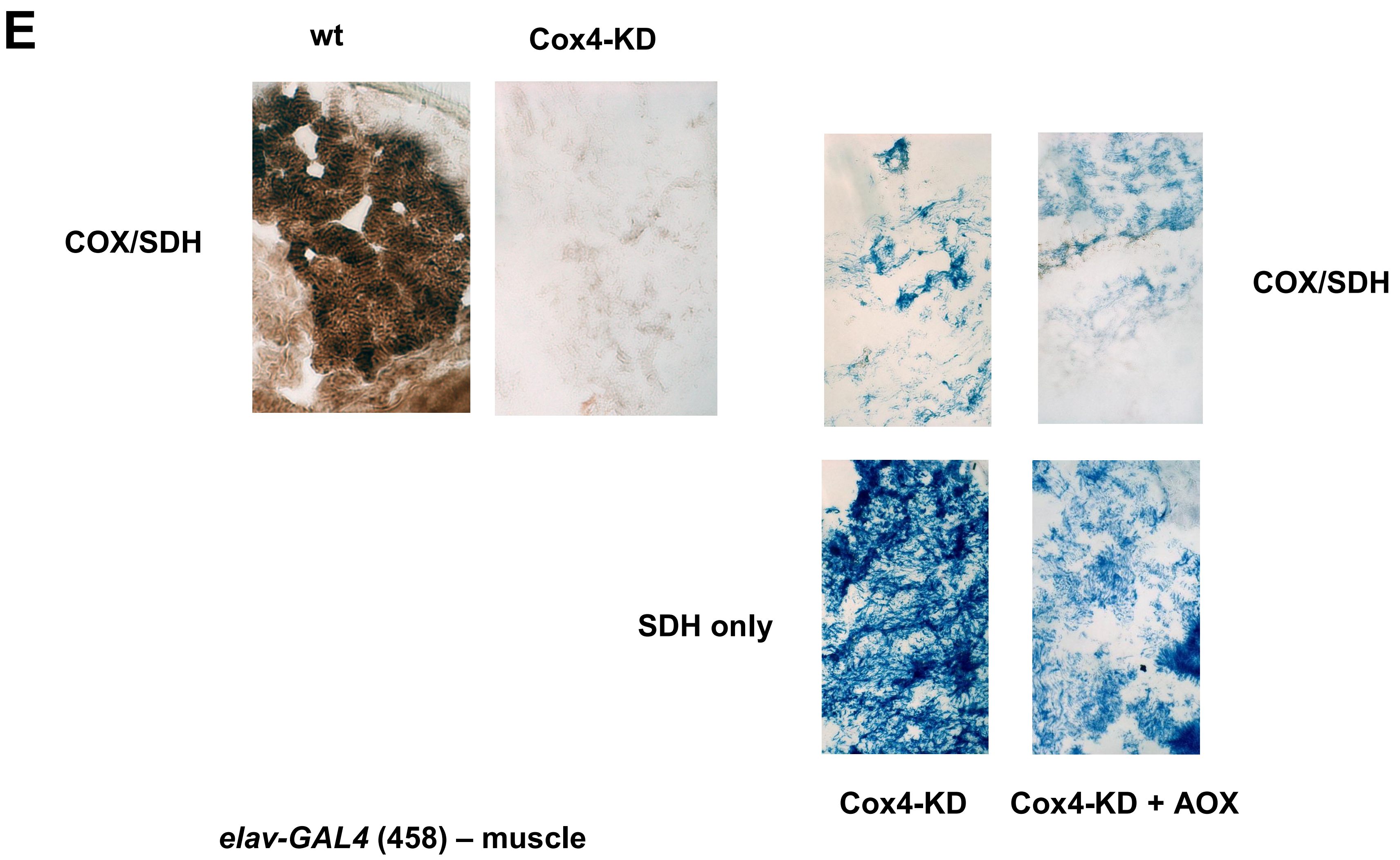
E
wt
Cox4-KD
COX/SDH
COX/SDH
SDH only
Cox4-KD
Cox4-KD + AOX
elav-GAL4 (458) – muscle

F

elav-GAL4 (8760) – muscle

COX only

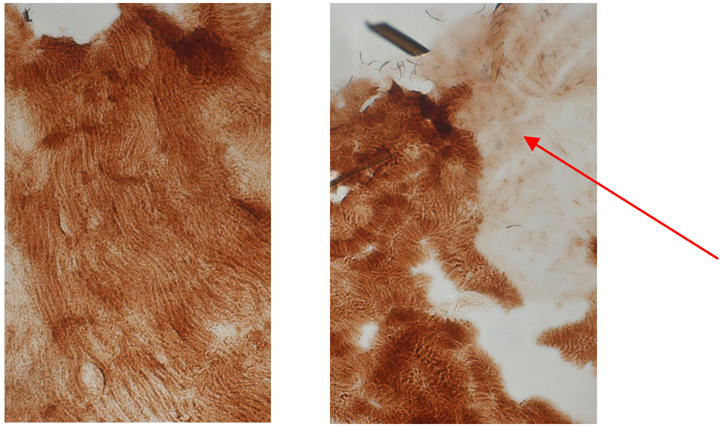

no RNAi Cox4-KD

G

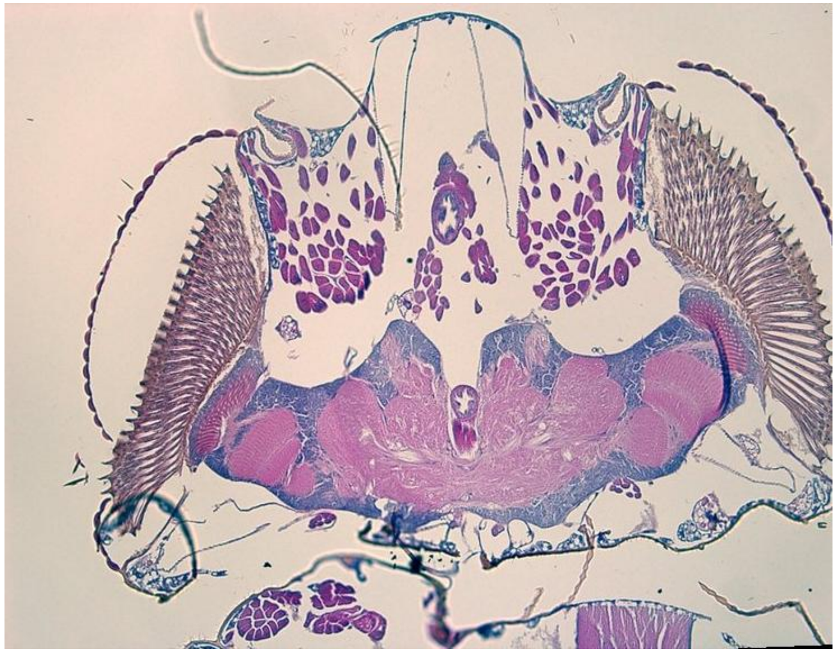

wild-type

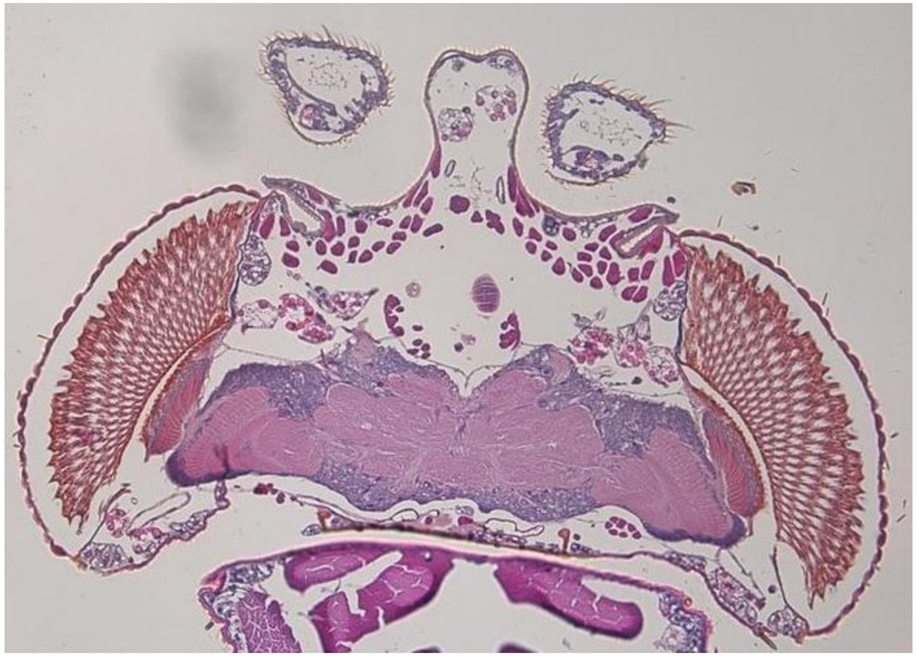

Cox4-KD / elav-GAL4 (458)

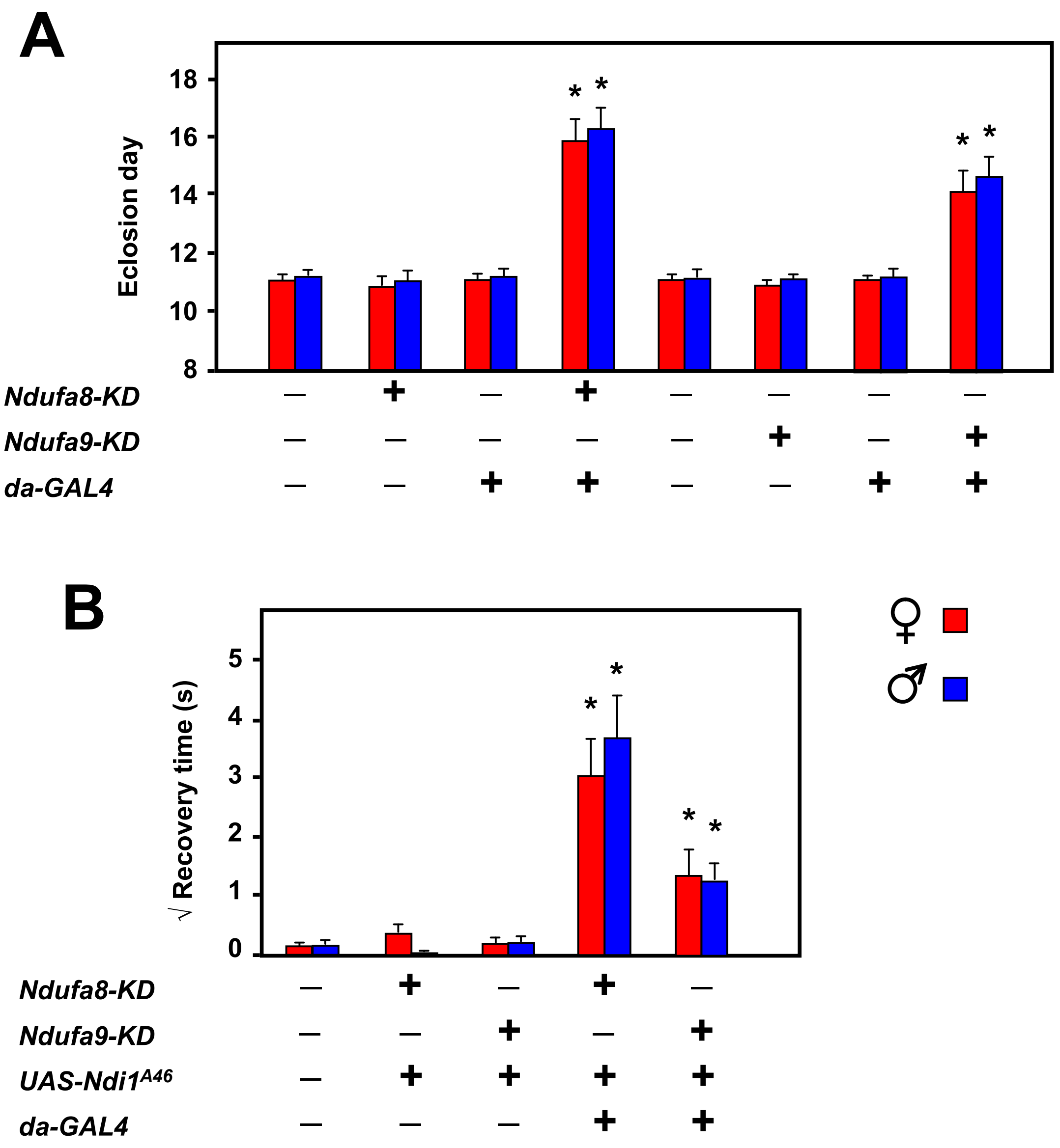

Figure S7, Kemppainen *et al* (page 1 of 2)

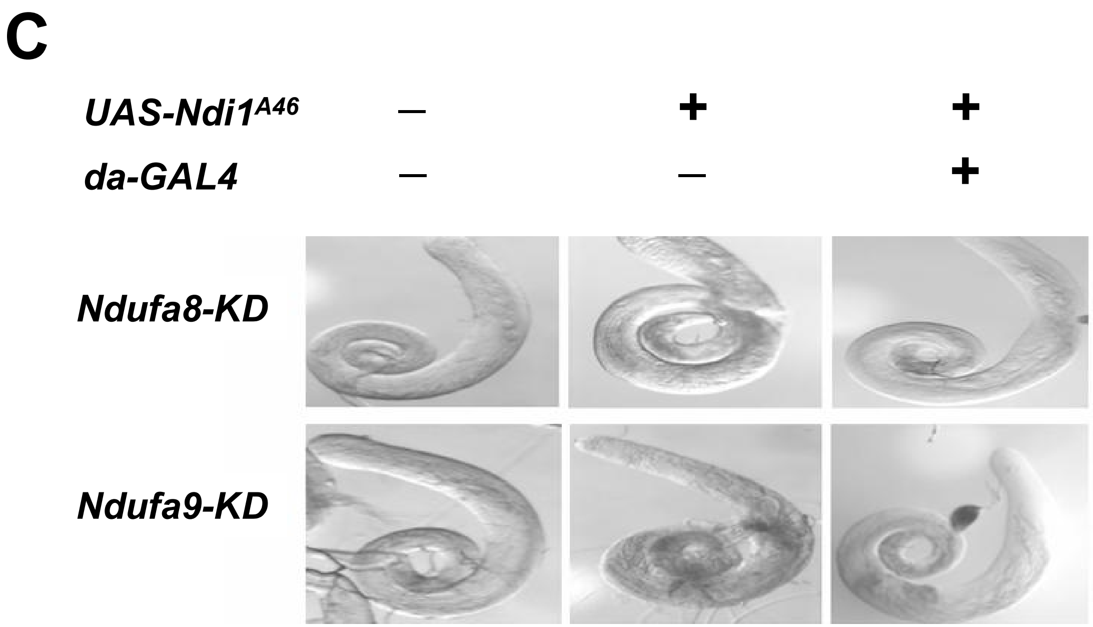

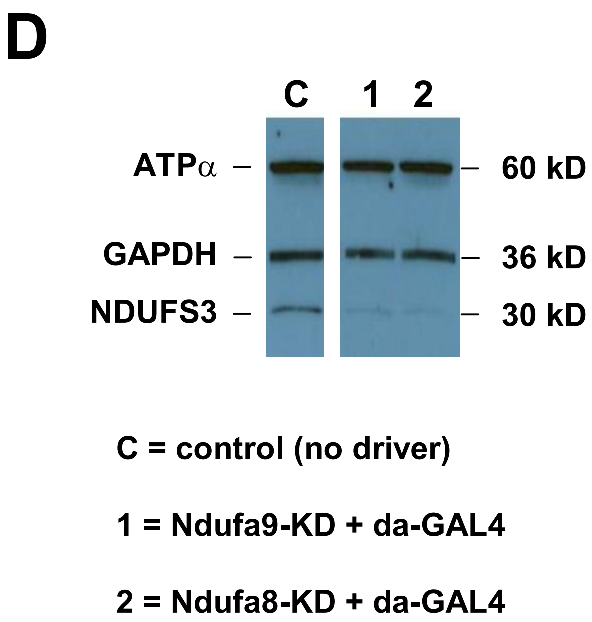

Figure S7, Kemppainen *et al* (page 2 of 2)

A

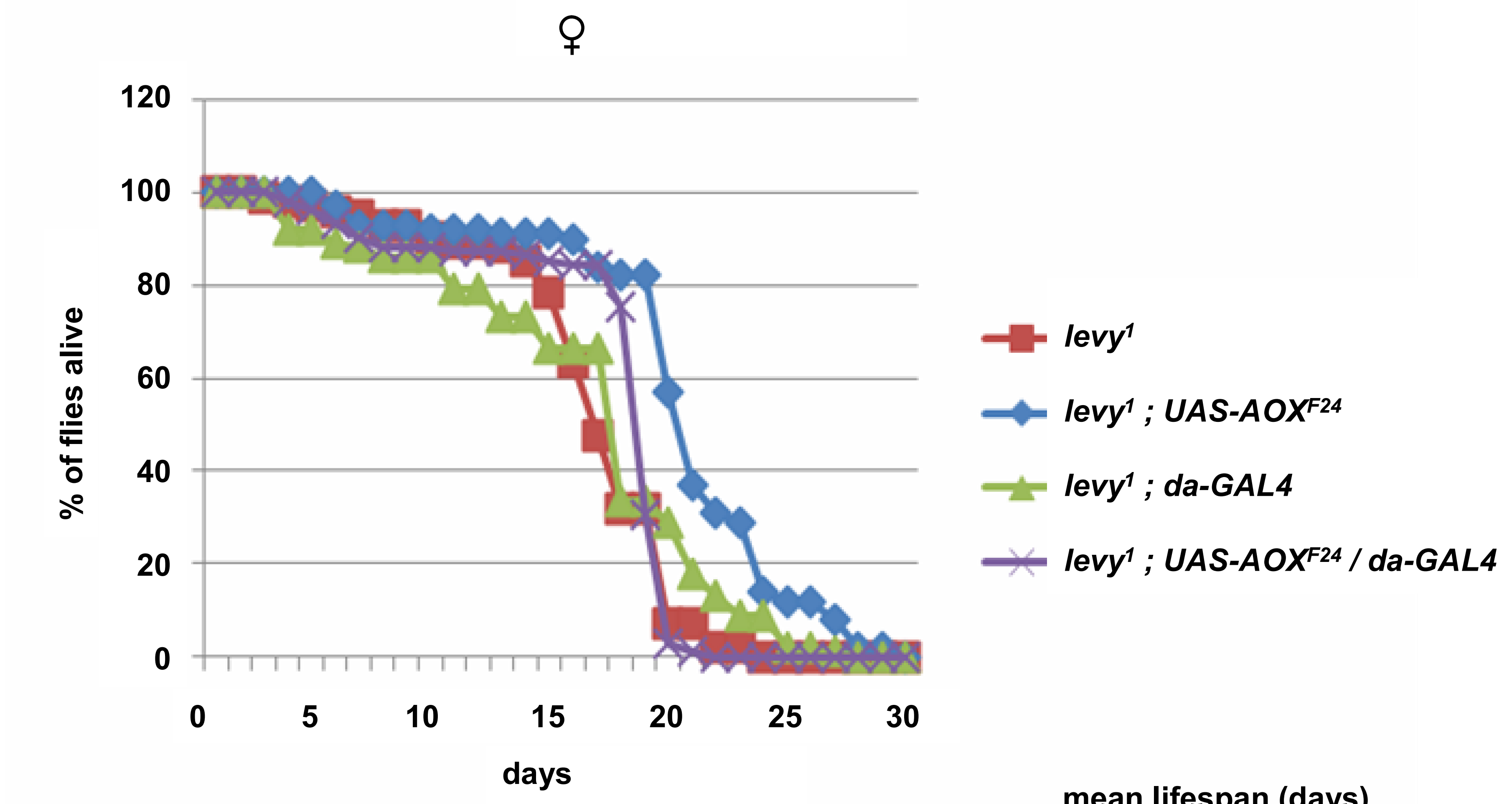

mean lifespan (days)

levy[1]	15.8
levy[1] *; UAS-AOX*^{F24}	16.0
levy[1] *; da-GAL4*	13.5
levy[1] *; UAS-AOX*^{F24} */ da-GAL4*	16.5

B

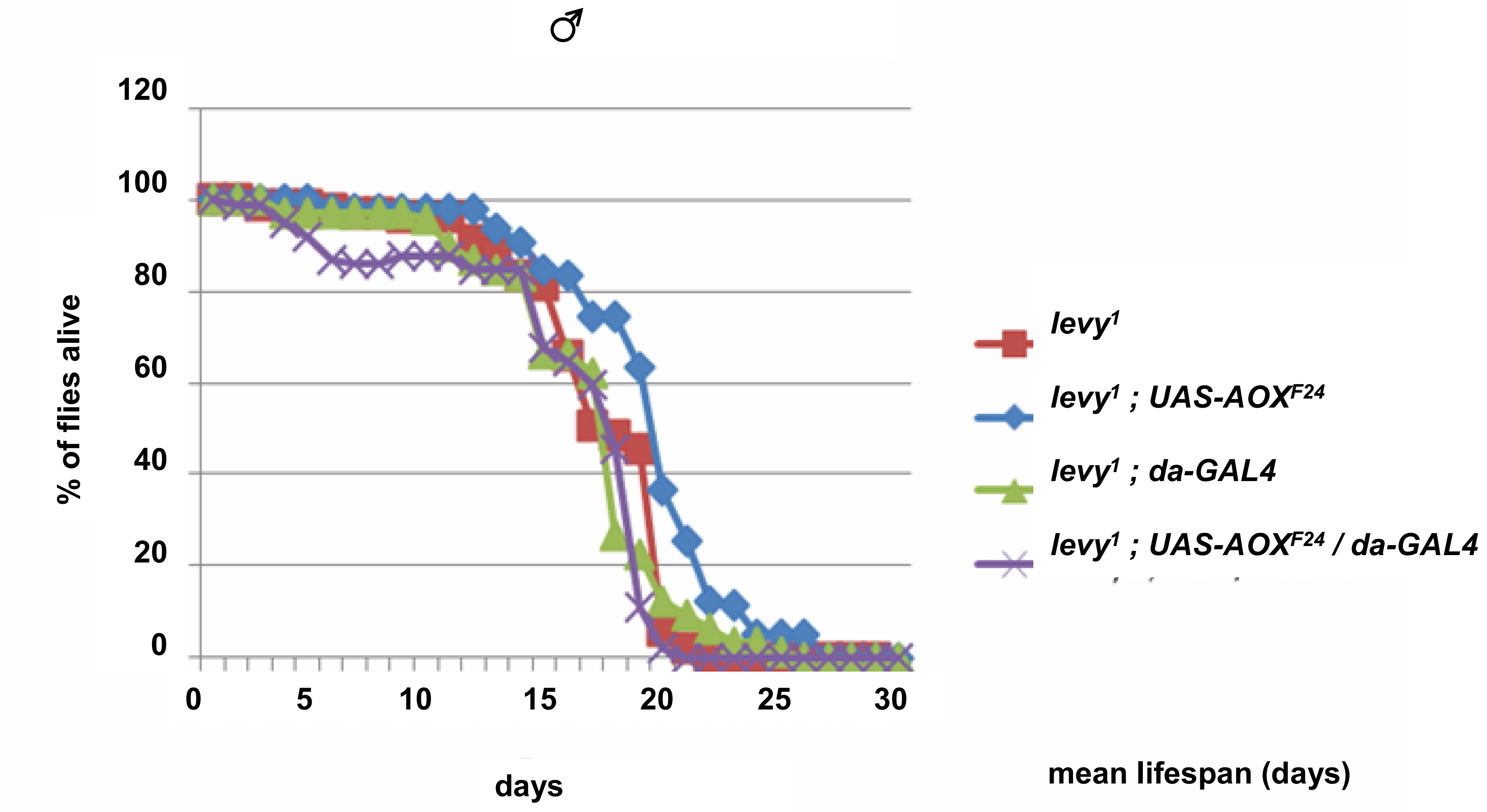

mean lifespan (days)

levy[1]	16.4
levy[1] ; UAS-AOX^F24	17.2
levy[1] ; da-GAL4	15.3
levy[1] ; UAS-AOX^F24 / da-GAL4	15.1

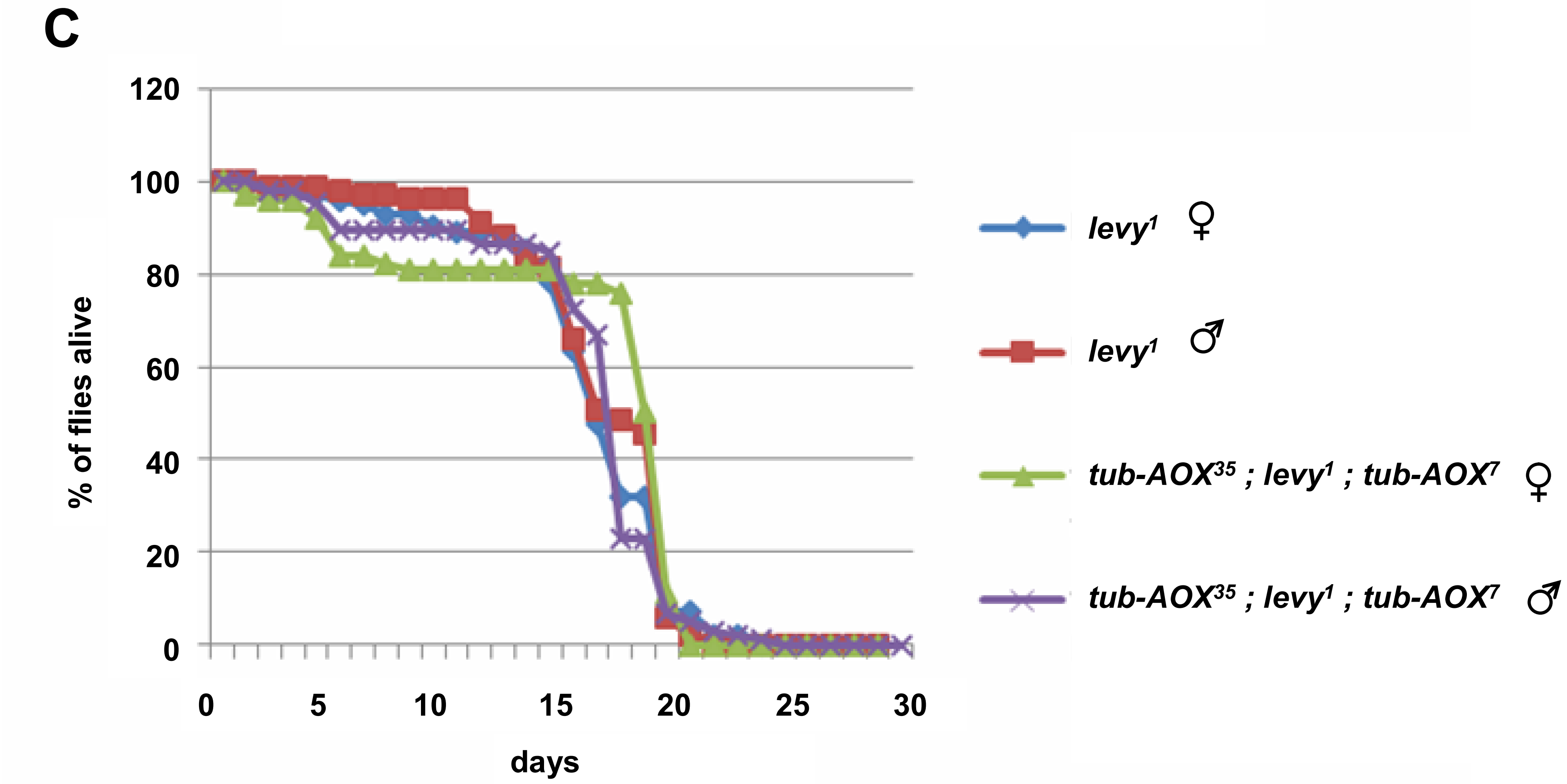

mean lifespan (days)

levy[1] F	15.8
levy[1] M	16.4
tub-AOX[35] ; levy[1] ; tub-AOX[7] F	15.9
tub-AOX[35] ; levy[1] ; tub-AOX[7] M	15.5

D

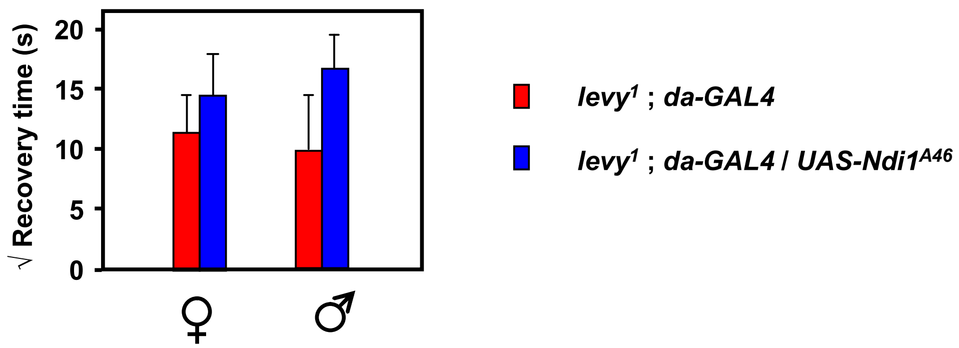

√ Recovery time (s)
20
15
10
5
0
♀
♂
levy1 ; da-GAL4
levy1 ; da-GAL4 / UAS-Ndi1A46

E

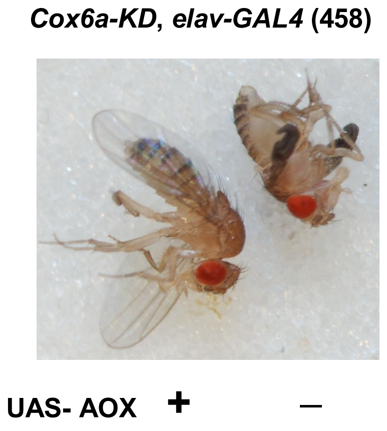

Cox6a-KD, elav-GAL4 (458)
UAS- AOX
+
–

TABLE S1

AOX rescue of *elav-GAL4* (Bloomington strain 458)-driven knockdown of *CoIV* or *CoVb*

Genotype	Sex	Typical Phenotype
elav-GAL4 / Y ; CoIV-KD / 2 ; 3 / 3	Male	Lethal; died as pupae or failed to eclose[#]
elav-GAL4 / Y ; CoIV-KD / 2 ; UAS-Ndi1^{A46} */ 3*	Male	Lethal; died as pupae or failed to eclose
elav-GAL4 / Y ; CoIV-KD / 2 ; UAS-AOX^{F6} */ 3*	Male	Eclosed, but very weak, died in food[#]
elav-GAL4 / X ; CoIV-KD / 2 ; 3 / 3	Female	Lethal; very few eclosed, all very weak[#]
elav-GAL4 / X ; CoIV-KD / 2 ; UAS-Ndi1^{A46} */ 3*	Female	Lethal; very few eclosed, all very weak
elav-GAL4 / X ; CoIV-KD / 2 ; UAS-AOX^{F6} */ 3*	Female	Eclosed and some inflated wings, but very weak, died in food[#]
elav-GAL4 / Y ; 2 / 2 ; CoVb-KD / 3	Male	Very weak, most died in food
elav-GAL4 / Y ; UAS-Ndi1^{B20} */ 2 ; CoVb-KD / 3*	Male	Very weak, most died in food
elav-GAL4 / Y ; UAS-AOX^{F24} */ 2 ; CoVb-KD / 3*	Male	Eclosed in substantial numbers and were viable
elav-GAL4 / X ; 2 / 2 ; CoVb-KD / 3	Female	Eclosed in substantial numbers, some died in food
elav-GAL4 / X ; UAS-AONdi1^{B20} */ 2 ; CoVb-KD* / 3	Female	Eclosed in substantial numbers, some died in food
elav-GAL4 / X ; UAS-AOX^{F24} */ 2 ; CoVb-KD / 3*	Female	Eclosed in normal numbers and were viable

[#]See Fig. S4F for examples of these phenotypes
